# Prior treatment status: impact on the efficacy and safety of teriflunomide in multiple sclerosis

**DOI:** 10.1186/s12883-020-01937-4

**Published:** 2020-10-06

**Authors:** Giancarlo Comi, Mark S. Freedman, José E. Meca-Lallana, Patrick Vermersch, Byoung Joon Kim, Alexander Parajeles, Keith R. Edwards, Ralf Gold, Houari Korideck, Jeffrey Chavin, Elizabeth M. Poole, Patricia K. Coyle

**Affiliations:** 1 Ospedale San Raffaele, Via Olgettina 58, 20132 Milan, Italy; 2 University of Ottawa and The Ottawa Hospital Research Institute, 501 Smyth Rd, Box, Ottawa, ON 601 Canada; 3 National Multiple Sclerosis Reference Center (CSUR), Hospital Virgen de la Arrixaca (IMIB-Arrixaca), Ctra, Madrid-Cartagena, s/n, 30120 Murcia, Spain; 4 Cátedra de Neuroinmunología Clínica y Esclerosis Múltiple, UCAM Universidad Católica San Antonio de Murcia, Campus de los Jerónimos, 30107 Murcia, Guadalupe Spain; 5 Univ. Lille, INSERM UMR-S1172 - Lille Neuroscience et Cognition, CHU Lille, FHU Imminent, Lille, France; 6 Department of Neurology, Samsung Medical Center, Sungkyunkwan University School of Medicine, 81 Ilwon-ro, Gangnam-gu, Seoul, South Korea; 7 San Juan de Dios Hospital, Paseo Colón, Merced, San José, Costa Rica; 8 Multiple Sclerosis Center of Northeastern New York, 1182 Troy-Schenectady Rd, Ste 203, Latham, NY 12110 USA; 9 St Josef Hospital, Ruhr University Bochum, 5092414 Gudrunstrasse 56, D-44791 Bochum, Germany; 10 Sanofi, 500 Kendall Street, 6th Floor, Cambridge, MA 02142 USA; 11 Present address: Dana-Farber Cancer Institute, Boston, MA USA; 12 Present address: Bluebirdbio, Cambridge, MA USA; 13 Department of Neurology, Stony Brook University, HSC T12-020, Stony Brook, NY 11794-8121 USA

**Keywords:** Disease-modifying therapy, Multiple sclerosis, Relapse rate, Teriflunomide, Treatment history

## Abstract

**Background:**

In this pooled, post hoc analysis of a phase 2 trial and the phase 3 TEMSO, TOWER, and TENERE clinical trials, long-term efficacy and safety of teriflunomide were assessed in subgroups of patients with relapsing multiple sclerosis (MS) defined by prior treatment status.

**Methods:**

Patients were classified according to their prior treatment status in the core and core plus extension periods. In the core period, patients were grouped according to treatment status at the start of the study: treatment naive (no prior disease-modifying therapy [DMT] or DMT > 2 years prior to randomization), previously treated with another DMT (DMT > 6 to ≤24 months prior to randomization), and recently treated with another DMT (DMT ≤6 months prior to randomization). In the core plus extension period, patients were re-baselined to the time of starting teriflunomide 14 mg and grouped according to prior treatment status at that time point. Efficacy endpoints included annualized relapse rate (ARR), probability of confirmed disability worsening (CDW) over 12 weeks, and Expanded Disability Status Scale (EDSS) score. The incidence of adverse events was also assessed.

**Results:**

Most frequently received prior DMTs at baseline were glatiramer acetate and interferon beta-1a across treatment groups. Teriflunomide 14 mg significantly reduced ARR versus placebo in the core period, regardless of prior treatment status. In the core and extension periods, adjusted ARRs were low (0.193–0.284) in patients treated with teriflunomide 14 mg across all subgroups. Probability of CDW by Year 4 was similar across subgroups; by Year 5, the percentage of patients with 12-week CDW was similar in treatment-naive patients and patients recently treated with another DMT (33.9 and 33.7%, respectively). EDSS scores were stable over time in all prior-treatment subgroups. There were no new or unexpected safety signals. Limitations include selective bias due to patient attrition, variability in subgroup size, and lack of magnetic resonance imaging outcomes.

**Conclusions:**

The efficacy and safety of teriflunomide 14 mg was similar in all patients with relapsing MS, regardless of prior treatment history.

**Trial registration:**

Phase 2 trial core: NCT01487096; Phase 2 trial extension: NCT00228163; TEMSO core: NCT00134563; TEMSO extension: NCT00803049; TOWER: NCT00751881; TENERE: NCT00883337.

## Background

In the 1990s and early 2000s, interferon beta (IFNB) and glatiramer acetate (GA) were the only disease-modifying therapies (DMTs) available for patients with relapsing forms of multiple sclerosis (RMS) [1]. In the past decade, additional DMTs with varying mechanisms of action have been approved [2]. Injectable DMTs continue to be used as first-line therapies for their efficacy and safety profiles [1, 3]; however, breakthrough disease activity, suboptimal tolerability, and patient nonadherence may necessitate a switch to another therapy. Few data are available on treatment sequencing and whether prior treatment experience impacts efficacy and safety of the second DMT after a switch.

Teriflunomide is a once-daily oral immunomodulator approved for the treatment of RMS or relapsing-remitting MS, depending on the local label, in more than 80 countries, including the United States [4] and countries of the European Union [5]. As of 2019, more than 100,000 patients were being treated with teriflunomide, with a total real-world exposure of approximately 285,800 patient-years. The efficacy and safety of teriflunomide have been established in a phase 2 trial (core: NCT01487096; extension: NCT00228163) [6], and the phase 3 TEMSO (core: NCT00134563; extension: NCT00803049) [7], TOWER (NCT00751881) [8], and TENERE (NCT00883337) [9] clinical trials. In the phase 2 study, TEMSO, and TOWER, teriflunomide 14 mg significantly reduced annualized relapse rate (ARR) and confirmed disability worsening (CDW) compared with placebo [6–8]. In TENERE, adjusted ARRs with teriflunomide 14 mg were the same as those for IFNB-1a [9].

In a previous analysis on the effect of number of prior DMTs on teriflunomide efficacy in the TEMSO and TOWER core studies, patients treated with teriflunomide 14 mg had reduced ARR and risk of disability worsening, regardless of whether they received a prior DMT [10]. The aim of the present analysis was to expand on these findings by evaluating efficacy and safety of teriflunomide in the long-term, in subgroups of patients defined by prior treatment status and recency.

## Methods

### Patients and study design

In this post hoc analysis, data were pooled from the phase 2 and phase 3 TEMSO, TOWER, and TENERE clinical trials and their extension studies (trial registrations: phase 2 trial core, NCT01487096; phase 2 trial extension, NCT00228163; TEMSO core, NCT00134563; TEMSO extension, NCT00803049; TOWER, NCT00751881; TENERE, NCT00883337). Complete study designs for these trials have been reported previously [6–9] and are summarized briefly below. The randomized studies contributing to this analysis adhered to CONSORT reporting guidelines.

Patients were eligible to participate in the clinical trials if they had RMS, were aged ≥18 years (upper age limit: 55 years for TEMSO and TOWER, 65 years for the phase 2 study, no limit for TENERE), and had an Expanded Disability Status Scale (EDSS) score ≤ 5.5 (TEMSO/TOWER/TENERE) or ≤ 6 (phase 2 study). Further eligibility criteria were 1 clinical relapse in the preceding year (phase 2 study) [6], ≥1 relapses in the previous 1 year, or ≥ 2 relapses in the previous 2 years (TOWER) [8]; or ≥ 2 clinical relapses in the previous 2 years or 1 relapse during the preceding year, without relapses in the 60 days before randomization (TEMSO) [7]. In TENERE, patients could not have had a clinical relapse in the 30 days prior to randomization [9].

In the phase 2, TEMSO, and TOWER core studies, patients were randomized 1:1:1 to receive placebo, teriflunomide 7 mg, or teriflunomide 14 mg for up to 36 weeks, 108 weeks, or ≥ 48 weeks, respectively. Patients treated with teriflunomide continued their original dose (phase 2/TEMSO) or received teriflunomide 14 mg regardless of original dose (TOWER). Placebo-treated patients were reassigned to teriflunomide 7 mg or 14 mg (phase 2/TEMSO) or teriflunomide 14 mg (TOWER). In the TENERE core study, patients were randomized 1:1:1 to receive teriflunomide 7 mg, teriflunomide 14 mg, or subcutaneous IFNB-1a 44 μg for ≥48 weeks; all patients received teriflunomide 14 mg in the extension.

For each study, patients who received previous DMT were eligible to participate, with the following exceptions: in the phase 2 and TENERE studies, patients were not eligible to participate if they had received prior treatment with IFNB within 4 or 3 months prior to randomization, respectively, and in all trials, patients could not enroll if they had received prior or concomitant treatment with cladribine, mitoxantrone, or other immunosuppressants. Data were not available on reason for stopping or switching from previous DMT.

### Study endpoints

Efficacy endpoints included ARR, probability of disability worsening confirmed over 12 weeks, and EDSS score. Cumulative duration of teriflunomide exposure and occurrence of adverse events (AEs) were also assessed.

In the phase 2 study, a relapse was defined as the appearance of a new symptom or worsening of a previous symptom due to MS, lasting 48 h in the absence of fever, and preceded by a period of stability of at least 30 days [6]. In the phase 3 trials, confirmed relapses were defined as new or worsening symptoms lasting ≥24 h without fever; relapses required an increase of 1 point in each of 2 EDSS functional system scores, or 2 points in 1 EDSS functional system score (excluding bowel and bladder function and cerebral function), or an increase of ≥0.5 points in EDSS score from the previous clinically stable assessment [7–9]. CDW was defined as an increase from baseline of ≥1.0 point in EDSS score (or ≥ 0.5 points for patients with a baseline EDSS score ≥ 5.5) confirmed over ≥12 weeks.

In the analysis of core study data, patients were grouped according to prior MS DMT status at core study baseline: treatment naive (including those treated > 2 years prior to randomization), previously treated with another DMT (> 6 months to ≤24 months prior to randomization), and recently treated with another DMT (≤6 months prior to randomization). The recently treated group included patients who had received IFNB-1a in the core TENERE study who entered the extension and were treated with teriflunomide 14 mg; their first 108 weeks on teriflunomide in the extension study were included in the teriflunomide 14 mg core study data analysis.

In the analyses of the core plus extension period, baseline was considered the time that patients started teriflunomide 14 mg. Patients were grouped according to prior treatment status: treatment naive, previously treated with another DMT, and recently treated with another DMT (Table 1).

**Table 1:** Definitions of prior MS treatment groups (core and extension analysis)

Group	Definition	Maximum time point available
Treatment naive	Patients with no prior DMT or DMT > 2 years prior to randomization	Year 13
Previously treated with another DMT	Patients whose most recent prior DMT was discontinued > 6 months to 2 years prior to randomization	Year 13
Recently treated with another DMT	Patients whose most recent prior DMT was discontinued within 6 months prior to randomization (excluded patients who recently received teriflunomide 7 mg)	Year 12

*DMT* disease-modifying therapy

### Statistical analysis

Patients who received teriflunomide 7 mg during the core period were not considered in the analysis. Two patient populations were included: the modified intention-to-treat population for efficacy outcomes, in which patients who received one or more study doses were analyzed according to the treatment group to which they were randomized, and the safety population, in which patients were analyzed according to the treatment they actually received.

Adjusted ARRs were compared between patients receiving placebo or teriflunomide according to the subgroups of prior treatment status and were derived using a Poisson regression model adjusted for baseline EDSS stratum (< 3.5 vs ≥3.5), age, gender, region, time since first diagnosis of MS, number of relapses in the year prior, and study. The response variable was the total number of confirmed relapses with an onset between randomization date and last dose date. Log-transformed study duration was included as an offset variable. Probability of 12-week CDW was assessed using Kaplan-Meier estimates.

## Results

### Baseline demographics and disease characteristics

Of patients treated with placebo or teriflunomide 14 mg in the core period, 1244 were treatment naive, 270 were previously treated with another DMT, and 247 were recently treated with another DMT (Table 2). Most baseline demographics and disease characteristics were similar across subgroups, except for time since diagnosis of MS, time since first MS symptoms, and mean EDSS score, which were numerically higher in previously treated patients compared with the other subgroups (Table 2). Across all subgroups, most patients had received either GA (17.1–37.1%) or IFNB-1a (39.6–69.6%) as their most recent prior DMT.

**Table 2:** Baseline demographics and disease characteristics (core period; safety population)

	Treatment naive (***n*** = 1244)		Previously treated with another DMT (***n*** = 270)		Recently treated with another DMT (***n*** = 247)		Overall population (***N*** = 1702)^**a**^	
	Placebo (***n*** = 586)	Teriflunomide 14 mg (***n*** = 658)	Placebo (***n*** = 131)	Teriflunomide 14 mg (***n*** = 139)	Placebo (***n*** = 89)	Teriflunomide 14 mg (***n*** = 158)	Placebo (***N*** = 806)	Teriflunomide 14 mg (***N*** = 896)^**a**^
Age, mean (SD) years	38.8 (9.0)	37.9 (9.2)	37.5 (8.6)	37.9 (8.4)	36.4 (9.5)	39.0 (9.6)	38.3 (9.0)	38.0 (9.1)
Female gender, *n* (%)	414 (70.6)	458 (69.6)	105 (80.2)	103 (74.1)	65 (73.0)	114 (72.2)	584 (72.5)	634 (70.8)
White, *n* (%)	518 (88.5)^b^	595 (90.4)	124 (94.7)	131 (94.9)^c^	84 (94.4)	154 (97.5)	726 (90.2)^d^	821 (91.7)^e^
Time since diagnosis of MS, mean (SD) years	4.42 (5.73)	4.40 (5.55)^f^	6.83 (4.92)	8.00 (5.55)	5.85 (5.26)	6.18 (6.04)	4.97 (5.62)	5.24 (5.85)^e^
Time since first symptoms of MS, mean (SD) years	7.79 (7.28)	7.49 (6.74)^f^	9.26 (5.63)	10.90 (6.87)	8.79 (7.02)	9.39 (7.19)	8.14 (7.02)	8.26 (6.91)^e^
Number of relapses within past year, mean (SD)	1.40 (0.71)^g^	1.37 (0.69)^h^	1.42 (0.87)^i^	1.36 (0.81)^j^	1.49 (0.81)^k^	0.90 (0.88)^l^	1.42 (0.75)^m^	1.37 (0.71)^n^
Baseline EDSS score								
Mean (SD)	2.61 (1.37)	2.57 (1.33)	2.97 (1.32)	2.79 (1.37)	2.58 (1.37)	2.49 (1.38)	2.67 (1.37)	2.64 (1.34)
Median (min, max)	2.50 (0.0, 6.0)	2.50 (0.0, 6.5)	3.00 (0.0, 6.0)	2.50 (0.0, 6.0)	2.50 (0.0, 5.5)	2.00 (0.0, 6.5)	2.50 (0.0, 6.0)	2.50 (0.0, 6.5)
Last prior DMT,^o,p^ *n* (%)								
Fingolimod	0	1 (4.5)	1 (0.8)	3 (2.2)	0	0	1 (0.4)	4 (1.5)
GA	6 (27.3)	4 (18.2)	42 (32.1)	51 (36.7)	33 (37.1)	27 (17.1)	81 (33.5)	82 (31.5)
IFNB^q^	0	0	3 (2.3)	0	2 (2.2)	2 (1.3)	5 (2.1)	2 (0.8)
IFNB-1a	11 (50.0)	9 (40.9)	57 (43.5)	55 (39.6)	36 (40.4)	110 (69.6)	104 (43.0)	115 (44.2)
IFNB-1b	5 (22.7)	8 (36.4)	27 (20.6)	27 (19.4)	18 (20.2)	19 (12.0)	50 (20.7)	54 (20.8)
Natalizumab	0	0	1 (0.8)	3 (2.2)	0	0	1 (0.4)	3 (1.2)

The safety population included patients who were analyzed according to the treatment they actually received during the studies; ^a^Excludes a cohort of 59 patients who switched from IFNB-1a to teriflunomide 14 mg (TENERE study); ^b^*n* = 585; ^c^*n* = 138; ^d^*n* = 805; ^e^*n* = 895; ^f^*n* = 657; ^g^*n* = 521; ^h^*n* = 598; ^i^*n* = 112; ^j^*n* = 119; ^k^*n* = 86; ^l^*n* = 151; ^m^*n* = 719; ^n^*n* = 809; ^o^Treatment-naive subgroup (no prior DMT in the 2 years prior to randomization): placebo, *n* = 22; TFL 14 mg, *n* = 22; ^p^Last prior DMT data were based on a total of 242 placebo-treated patients and 260 teriflunomide 14 mg-treated patients for the overall population; ^q^IFNB formulation not specified. *DMT* disease-modifying therapy, *EDSS* Expanded Disability Status Scale, *GA* glatiramer acetate, *IFNB* interferon beta, *max* maximum, *min* minimum, *MS* minimum sclerosis, *SD* standard deviation, *TFL* teriflunomide

In the safety population from the pooled core and extension studies, there were 1339 patients exposed to teriflunomide 14 mg, of whom 1018 were treatment naive (mean [standard deviation] exposure: 196.1 [148.3] weeks), 163 were previously treated (187.4 [161.4] weeks), and 158 were recently treated (160.7 [115.0] weeks) (Table 3). Patient baseline characteristics were similar across the subgroups at the time of starting teriflunomide 14 mg, except previously treated patients had numerically higher mean time since MS diagnosis, time since first symptoms of MS, number of relapses within the past year, and mean EDSS score compared with the other subgroups.

**Table 3:** Baseline characteristics at start of teriflunomide 14 mg (core and extension period; safety population)

	Treatment naive (***n*** = 1018)	Previously treated with another DMT (***n*** = 163)	Recently treated with another DMT (***n*** = 158)	Overall Population (***N*** = 1696)^**a**^
Age, mean (SD) years	38.64 (9.06)	37.82 (8.24)	38.97 (9.62)	38.68 (9.12)
Female gender, *n* (%)	718 (70.5)	125 (76.7)	114 (72.2)	1211 (71.4)
White, *n* (%)	918 (90.3)^b^	154 (95.1)^c^	154 (97.5)	1536 (90.7)^d^
Time since diagnosis of MS, mean (SD) years	5.06 (5.44)^b^	7.95 (5.38)	6.18 (6.04)	5.80 (5.60)^e^
Time since first symptoms of MS, mean (SD) years	8.20 (6.71)^b^	10.72 (6.69)	9.39 (7.19)	8.90 (6.87)^e^
Number of relapses within past year, mean (SD)	1.00 (0.82)^f^	1.22 (0.85)^g^	0.90 (0.88)^h^	0.87 (0.84)^i^
Baseline EDSS score				
Mean (SD)	2.59 (1.43)	2.74 (1.40)	2.49 (1.38)	2.58 (1.43)
Median (min, max)	2.50 (0.0, 8.0)	2.50 (0.0, 6.5)	2.00 (0.0, 6.5)	2.50 (0.0, 8.0)
Last prior DMT,^j,k^ *n* (%)				
Fingolimod	2 (1.8)	3 (1.8)	0	5 (0.6)
GA	36 (33.0)	58 (35.6)	27 (17.1)	121 (15.4)
IFNB^l^	1 (0.9)	0	2 (1.3)	3 (0.4)
IFNB-1a	39 (35.8)	68 (41.7)	110 (69.6)	217 (27.6)
IFNB-1b	31 (28.4)	31 (19.0)	19 (12.0)	81 (10.3)
Natalizumab	0	3 (1.8)	0	3 (0.4)
Teriflunomide 7 mg	0	0	0	357 (45.4)

^a^Includes an additional cohort of 357 patients from the TOWER and TENERE studies who switched from teriflunomide 7 mg to 14 mg in the extension period; ^b^*n* = 1017; ^c^*n* = 162; ^d^*n* = 1694; ^e^*n* = 1695; ^f^*n* = 958; ^g^*n* = 143; ^h^*n* = 151; ^i^*n* = 1609; ^j^Data reported in the treatment-naive subgroup include patients with DMT use > 2 years prior to receiving teriflunomide 14 mg (*n* = 109); ^k^Last prior DMT data were based on a total of 787 patients for the overall population; ^l^IFNB formulation not specified; *DMT* disease-modifying therapy, *EDSS* Expanded Disability Status Scale, *GA* glatiramer acetate, *IFNB* interferon beta, *max* maximum, *min* minimum, *MS* multiple sclerosis, *SD* standard deviation

### Efficacy

**Fig. 1:**
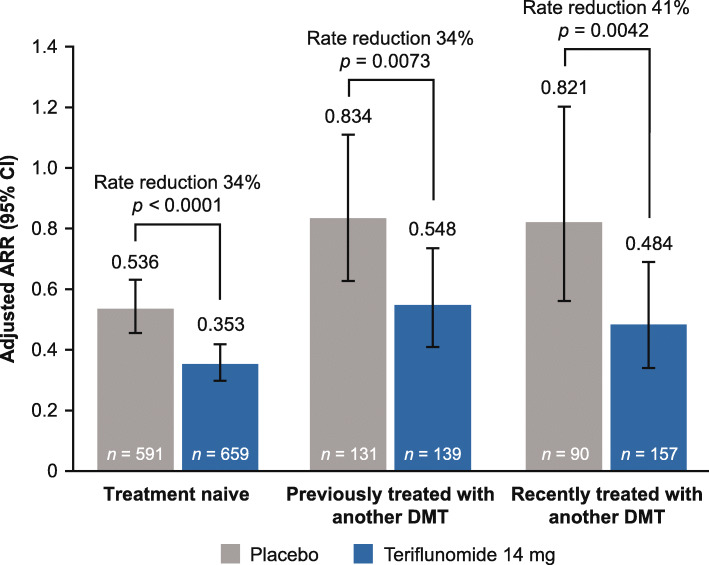
Adjusted ARR with teriflunomide 14 mg or placebo, stratified by prior treatment history (core period) in the modified intent-to-treat population. The core period for the recently-treated-with-another-DMT group included patients who received IFNB-1a in the core TENERE study who entered the extension and were treated with teriflunomide 14 mg; their first 108 weeks on teriflunomide in the extension study were included. The modified intent-to-treat population included patients who received one or more study doses and were analyzed according to the treatment group to which they were randomized. *ARR* annualized relapse rate, *CI* confidence interval, *DMT* disease-modifying therapy, *IFNB* interferon beta

In the core period, overall adjusted ARR reduction for teriflunomide 14 mg versus placebo was 34% (*p* < 0.0001) in treatment-naive patients, 34% (*p* = 0.0073) in previously treated patients, and 41% (*p* = 0.0042) in recently treated patients (Fig. 1).

**Fig. 2:**
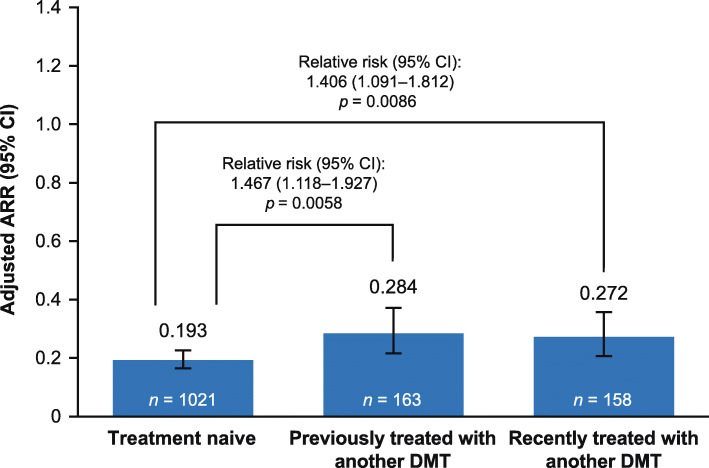
Overall adjusted ARR in patients treated with teriflunomide 14 mg (core and extension period). *ARR* annualized relapse rate, *CI* confidence interval, *DMT* disease-modifying therapy

In the core and extension period, overall adjusted ARRs (95% confidence interval [CI]) for teriflunomide 14 mg were 0.193 (0.165–0.226) in treatment-naive patients, 0.284 (0.216–0.372) in previously treated patients, and 0.272 (0.207–0.357) in recently treated patients (Fig. 2).

**Fig. 3:**
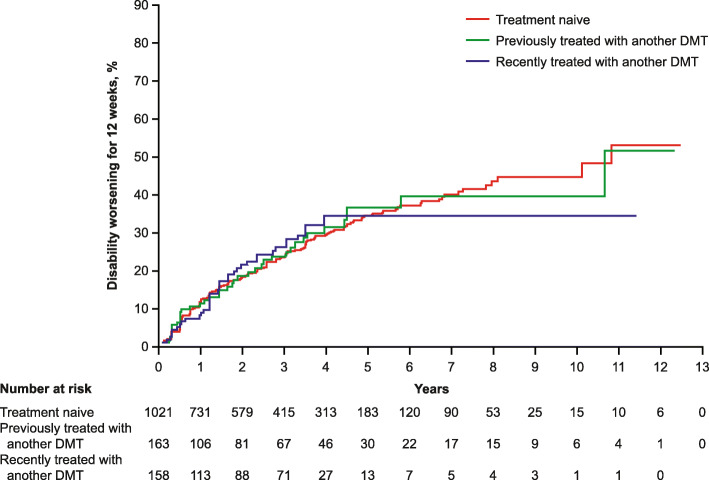
Percentage of teriflunomide 14 mg-treated patients with 12-week CDW (core and extension period). *CDW* confirmed disability worsening, *DMT* disease-modifying therapy

The percentage of patients with 12-week CDW (95% CI) was 52.7% (41.3–65.1%) in treatment-naive patients at Year 13, 51.2% (30.9–75.1%) in patients previously treated with another DMT at Year 13, and 33.7% (25.0–44.4%) in patients recently treated with another DMT at Year 12; however, the number of patients analyzed at the end of the trial period was low (Fig. 3). At Year 5, the maximum time point at which each treatment subgroup had at least 10 patients, the percentage of patients with 12-week CDW was 33.9% (30.1–37.9%) in treatment-naive patients, 36.2% (26.9–47.4%) in previously treated patients, and 33.7% (25.0–44.4%) in recently treated patients (Fig. 3).

EDSS scores were stable across all treatment subgroups over the core and extension period. At the start of teriflunomide 14 mg treatment, mean (95% CI) EDSS scores were 2.58 (2.50–2.67) in treatment-naive patients (*n* = 1020), 2.74 (2.52–2.96) in previously treated patients (*n* = 163), and 2.48 (2.26–2.69) in recently treated patients (*n* = 158). At Year 8, the maximum time point at which each subgroup had at least 10 patients, mean (95% CI) EDSS scores were 2.91 (2.65–3.17) in treatment-naive patients (*n* = 150), 2.58 (1.84–3.32) in patients previously treated with another DMT (*n* = 24), and 3.64 (2.20–5.07) in patients recently treated with another DMT (*n* = 11).

### Safety

In the core and extension periods, the percentages of patients with AEs and serious AEs were comparable across subgroups (90.2–96.3% and 19.3–24.5%, respectively; Table 4). The occurrence of AEs leading to permanent treatment discontinuation was slightly lower in the treatment-naive subgroup (15.2%; Table 4).

**Table 4:** Occurrence of AEs with teriflunomide 14 mg^a^ (core and extension period)

	Treatment naive (***n*** = 1018)	Previously treated with another DMT (***n*** = 163)	Recently treated with another DMT (***n*** = 158)	Overall population (***N*** = 1696)^**b**^
Patients with any AE, *n* (%)	918 (90.2)	157 (96.3)	145 (91.8)	1503 (88.6)
Patients with any SAE, *n* (%)	196 (19.3)	40 (24.5)	34 (21.5)	312 (18.4)
Patients with any AE leading to death, *n* (%)	7 (0.7)	0	0	10 (0.6)
Patients with any AE leading to permanent treatment discontinuation, *n* (%)	155 (15.2)	33 (20.2)	31 (19.6)	244 (14.4)
AEs with incidence > 10% in any prior DMT subgroup, *n* (%)				
Nasopharyngitis	249 (24.5)	47 (28.8)	36 (22.8)	391 (23.1)
ALT increase	185 (18.2)	25 (15.3)	18 (11.4)	246 (14.5)
Headache	185 (18.2)	32 (19.6)	40 (25.3)	296 (17.5)
Diarrhea	178 (17.5)	41 (25.2)	35 (22.2)	284 (16.7)
Hair thinning (alopecia)	157 (15.4)	28 (17.2)	25 (15.8)	226 (13.3)
Back pain	148 (14.5)	34 (20.9)	21 (13.3)	231 (13.6)
Fatigue	136 (13.4)	26 (16.0)	25 (15.8)	203 (12.0)
Influenza	133 (13.1)	26 (16.0)	17 (10.8)	200 (11.8)
Nausea	121 (11.9)	26 (16.0)	21 (13.3)	184 (10.8)
Pain in extremity	121 (11.9)	24 (14.7)	14 (8.9)	181 (10.7)
Upper respiratory tract infection	121 (11.9)	31 (19.0)	23 (14.6)	202 (11.9)
Urinary tract infection	113 (11.1)	29 (17.8)	21 (13.3)	197 (11.6)
Paresthesia	102 (10.0)	24 (14.7)	15 (9.5)	157 (9.3)
Hypoesthesia	99 (9.7)	22 (13.5)	14 (8.9)	148 (8.7)
Hypertension	94 (9.2)	18 (11.0)	21 (13.3)	157 (9.3)
Arthralgia	93 (9.1)	22 (13.5)	14 (8.9)	147 (8.7)
Bronchitis	78 (7.7)	18 (11.0)	15 (9.5)	126 (7.4)
Sinusitis	68 (6.7)	24 (14.7)	16 (10.1)	126 (7.4)
Fall	67 (6.6)	20 (12.3)	17 (10.8)	120 (7.1)
Abdominal pain, upper	60 (5.9)	10 (6.1)	19 (12.0)	98 (5.8)
Insomnia	56 (5.5)	20 (12.3)	12 (7.6)	94 (5.5)
Gastroenteritis	50 (4.9)	18 (11.0)	13 (8.2)	94 (5.5)

^a^Data are reported for AEs occurring after patients started teriflunomide 14 mg; ^b^Includes an additional cohort of 357 patients from the TOWER and TENERE studies who switched from teriflunomide 7 mg to 14 mg in the extension period; *AE* adverse event, *ALT* alanine aminotransferase, *DMT* disease-modifying therapy, *SAE* serious adverse event

For the treatment-naive, previously treated, and recently treated subgroups, the incidence rates (per 1000 person-years) of any AE in the core period were 584, 610, and 456, respectively. By Year 4, respective incidence rates were 327, 386, and 351. In the core and extension period, the AE incidence rates were 240, 268, and 298.

There were no new or unexpected safety findings with teriflunomide 14 mg in any of the subgroups of prior treatment status. The most frequently reported AEs included nasopharyngitis, alanine aminotransferase increase, headache, diarrhea, and hair thinning (alopecia). Seven deaths were reported, all in the treatment-naive group, and were due to pulmonary tuberculosis and suicide (both reported as potentially related to teriflunomide), and tachycardia, acute cardiac failure, gastrointestinal hemorrhage, suicide, and septicemia (none deemed to be related to teriflunomide).

## Discussion

In this post hoc analysis, ARRs were significantly lower with teriflunomide 14 mg versus placebo in the core studies and remained low in the core and extension periods in patients who were treatment naive, previously treated with another DMT, or recently treated with another DMT. Although prior treatment status did not affect teriflunomide treatment effect versus placebo, treatment-experienced patients overall had higher ARRs than those who were treatment naive. This may be explained by the inclusion criterion for active disease at core study baseline, selecting for patients with suboptimal DMT response in the treatment-experienced subgroups. However, prior treatment status did not affect disability outcomes after teriflunomide initiation. At Year 5, 12-week CDW was similar across all subgroups, and EDSS scores remained stable through Year 8. These results suggest that regardless of treatment history, teriflunomide 14 mg is efficacious in the short- and long-terms.

Long-term efficacy of teriflunomide may be related to its mechanism of action. Teriflunomide is an inhibitor of de novo pyrimidine biosynthesis, through a mechanism that also decreases oxidative phosphorylation. High-affinity T cells have greater dependence than lower-affinity T cells on oxidative phosphorylation to supply energy; therefore, teriflunomide may have a selective antiproliferative effect on high-affinity autoimmune T-cell clones. This effect may both shape the T-cell receptor repertoire (by preventing expansion of autoimmune clones) and decrease autoimmune T-cell levels relative to inducible regulatory T cells [11].

The safety data observed in this pooled post hoc analysis were consistent with previous findings; no new or unexpected safety findings were observed. The incidence rate of AEs in the core plus extension period was higher when analyzed through Year 4 compared with through the end of the trial period, suggesting that most AEs occur relatively early after teriflunomide initiation and decline as treatment continues. Regarding the deaths in the study, signal assessments, safety governance, and medical evaluations have not established a causative role of teriflunomide in the fatal outcomes, but rather a number of other causes were plausible.

Several limitations are associated with this study. As in all extension studies, there was a progressive loss of evaluable patients over the duration of the follow-up periods across all subgroups. Thus efficacy outcomes could be biased by the selective loss of poor responders, although patient attrition over time was also due to variable study durations and the timing of teriflunomide availability in each country. The number of patients also varied within each subgroup. Additional magnetic resonance imaging (MRI) data, including T2 hyperintense lesions and brain atrophy, would further clarify the effect of prior treatment status on teriflunomide efficacy.

## Conclusions

Across prior DMT subgroups, treatment with teriflunomide 14 mg produced similar treatment effect on relapses, with stable disability, and no apparent effect on tolerability and safety. These data may help to guide treatment decision making in the clinical setting, particularly in treatment-naive patients or those who do not respond to older first-line therapies.

## Supplementary information

**Additional file 1: Supplementary Table 1.** List of Institutional Review Boards and Independent Ethics Committees that approved the procedures at each participating site for the phase 2 clinical trial (A), and phase 3 TEMSO (B), TOWER (C), and TENERE (D) clinical trials.

## Data Availability

Qualified researchers may request access to patient-level data and related study documents including the clinical study report, study protocol with any amendments, blank case report form, statistical analysis plan, and dataset specifications. Patient-level data will be anonymized and study documents will be redacted to protect the privacy of the trial participants. Further details on Sanofi’s data-sharing criteria, eligible studies, and process for requesting access can be found at https://www.clinicalstudydatarequest.com.

## Abbreviations

AE: Adverse event
ARR: Annualized relapse rate
CDW: Confirmed disability worsening
CI: Confidence interval
DMT: Disease-modifying therapy
EDSS: Expanded Disability Status Scale
GA: Glatiramer acetate
IFNB: Interferon beta
MRI: Magnetic resonance imaging
MS: Multiple sclerosis
RMS: Relapsing forms of multiple sclerosis
