# Supplementary material for: Prior treatment status: impact on the efficacy and safety of teriflunomide in multiple sclerosis
Source: BMC Neurol. 2020 Oct 6;20:364. doi: 10.1186/s12883-020-01937-4 (PMC7539502; doi:10.1186/s12883-020-01937-4)
Supplement: Supplementary file 1 — Additional file 1: Supplementary Table 1. List of Institutional Review Boards and Independent Ethics Committees that approved the procedures at each participating site for the phase 2 clinical trial (A), and phase 3 TEMSO (B), TOWER (C), and TENERE (D) clinical trials. [file 12883_2020_1937_MOESM1_ESM.docx]

**Supplementary information**

**Supplementary Table 1.** List of Institutional Review Boards and Independent Ethics Committees that approved the procedures at each participating site for the phase 2 clinical trial (A), and phase 3 TEMSO (B), TOWER (C), and TENERE (D) clinical trials.

**A. Phase 2 trial.**

| **Institutional Review Board (IRB)/Independent Ethics Committee (IEC) Name and Address** |
| --- |
| Queen Elizabeth II Health Sciences Centre, 5790, University Avenue, Halifax NS B3H 1V7, Canada |
| Montreal Neurological Hospital, 3801, rue University, Montréal, QC H3A 2B4, Canada |
| CHUM, Hôtel-Dieu, 3840, rue Saint-Hurbain, Montréal QC H2W 1T8, Canada |
| Ottawa Hospital General Campus, 501, Smyth Road, Ottawa ON K1H 8L6, Canada |
| St Michael’s Hospital, 30, Bond Street Toronto ON M5B 1W8, Canada |
| University of Western Ontario, RM 00045 Dental Sciences Building, London ON N6A 5C1, Canada |
| University of Calgary, 3330, Hospital Drive N.W. Calgary Alberta T2N 4N1, Canada |
| University of British Columbia, 2211, Wesbrook Mall, Vancouver BC V6T 2B5, Canada |
| Hôpital de l’Enfant-Jésus, 1401, 18e Rue, Québec, QC G1J 1Z4, Canada |
| University of Manitoba, P126-770 Bannatyne Avenue, Winnipeg Manitoba R3E 0W3, Canada |
| CCPPRB of Lyon B (Comité Consultatif de Protection des personnes dans la Recherche Biomédicale), Porte 16, Hôpital Hôtel Dieu. 1, Place de l’Hôpital, 69288 Lyon Cedex 02, France |

**B. TEMSO.**

| **Institutional Review Board (IRB)/Independent Ethics Committee (IEC) Name and Address** | | | |
| --- | --- | --- | --- |
| **Local IRB/IEC** | | **National IRB/IEC** | |
| Capital Health Research Ethics Board  5790 University Avenue,  Room 118  Halifax, NS B3H 1V7 CANADA | |  | |
| Comité d'Éthique de la recherche de l'Hôpital Enfant-Jésus du CHA 1401 - 18ième Rue Québec, QC G1J 1Z4  CANADA | |  | |
| Comité d'Éthique de la recherche de l'Hôpital St-Luc  Edifice Cooper  3981 Boul. St-Laurent, M207  Montreal, QC H2W 1Y5  CANADA | |  | |
| Ottawa Hospital Research Ethics Board 751 Parkdale Avenue,  Suite 106  Ottawa, ON K1Y 1J7 CANADA | |  | |
| Comité d'Éthique de la recherche  Hôpital Charles LeMoyne  3120, Boulevard Taschereau  Greenfield Park, QC J2V 2H1  CANADA | |  | |
| The University of Western Ontario Office of Research Ethics 4180 Support Services Building  London, ON N6A 5C1 Canada | |  | |
| Bio Medical Research Ethics Board  Falculty of Medicine University of Manitoba P126 - 770 Bannatyne Avenue  Winnipeg, MB R3E 0W3 CANADA | |  | |
| Conjoint Health Ethics Research Board Room 93, HMRB  3330 Hospital Dr N.W. Calgary, AB T2N 4N1 CANADA | |  | |
| St. Michael's Hospital Research Ethics Board 30 Bond Street Toronto, ON M5B 1W8  CANADA | |  | |
| University of British Columbia  Clinical Research Ethics Board  Room 210, 828 West 10th Avenue Vancouver, BC V5Z 1L8 CANADA | |  | |
| Human Investigation Committee  95 Bonaventure Avenue 2nd Floor, Eastern Trust Building  St. John's, NL A1B 2X5 CANADA | |  | |
| Comité d’éthique de la recherche  500, Boul. de l’Hôpital Bureau 202 Gatineau, Quebec J8V 2P5  CANADA | | Canadian Shield Ethics Review Board  501 Deerhurst Drive  Suite 102  Burlington, ON L7L 5T1 CANADA | |
|  | | Schulman Associates Institutional Review Board, Inc., 4290 Glendale-Milford Road, Cincinnati, OH 45242  USA | |
| Human Investigation Committee, Wayne State University, 101 East Alexandrine Building, Detroit, MI 48201  USA | |  | |
| Lehigh Valley Hospital IRB, 17th & Chew Streets 6th floor, Allentown, PA 18102  USA | |  | |
|  | | Schulman Associates Institutional Review Board, Iinc., 4290 Glendale-Milford Road, Cincinnati, OH 45242  USA | |
| Human Subjects Committee, University of Kansas Medical Center, 3901 Rainbow boulevard, Kansas City,  KS 66160 USA | |  | |
|  | | Schulman Associates Institutional Review Board, inc., 4290 Glendale-Milford road, Cincinnati, oh 45242  USA | |
|  | | Schulman Associates Institutional Review Board, inc., 4290 Glendale-Milford road,  Cincinnati, oh 45242 USA | |
|  | Ethics Review Committee on Human Research of the University of Tartu Office of Research and Institutional Development  Ulikooli 18 50090 Tartu  ESTONIA | | |
|  | Ethics Review Committee on Human Research of the University of Tartu Office of Research and Institutional  Develooment  Ulikooli 18  50090 Tartu ESTONIA | | |
| Ethikkommisson der | Ethikkommission der | | |
| Stadt Wien | Medizinischen | | |
| Thomas-Klesitl-Platz 8/2 | UniversitatGraz | | |
| 1030 Wien | Auenbruggerplatz 2 | | |
| AUSTRIA | 8036Graz | | |
|  | AUSTRIA | | |
| Ethikkommission | Ethikkommissionder | | |
| der Med. | Medizinischen | | |
| Universitat Wien | Universitat Graz | | |
| Borschkegasse | Auenbruggerplatz 2 | | |
| 8b/E06 | 8036 Graz | | |
| 1090 Wien | AUSTRIA | | |
| AUSTRIA |  | | |
| Ethikkommission der | Ethikkommisison der | | |
| Medizinischen | Medizinischen | | |
| UniversitatGraz | Universitat Graz | | |
| Auenbruggerplatz 2 | Auenbruggerplatz 2 | | |
| 8036 Graz AUSTRIA | 8036 Graz AUSTRIA | | |
| Ethikkommissionder Med. Universitat Innsbruck Geschaftsstelle der EK lnnrain 43  6020 Innsbruck AUSTRIA | Ethikkommission der Medizinischen Universitat Graz Auenbruggerplatz 2  8036 Graz AUSTRIA | | |
| Ethikkommissionbeider Basel *I* EKBB Hebelstrasse 53  4056 Basel SWITZERLAND |  | | |
| Ruhr-Universitat Bochum  Ethik-Kommission Biirckle-de-la-Camp Platz 1  44789 Bochurn  GERMANY |  | | |
| Landesarztekammer Berlin Ethik-Kommission Friedrichstr. 16  10969 Berlin  GERMANY |  | | |
| Landesatzekammer- Rheinland-Pfalz Deutschhausplatz 3  55019 Mainz  GERMANY |  | |  |
| Landesarztekammer Hessen  Im Vogelgesang 3  60488 Frankfurt/Main GERMANY |  | |  |
| Universitatskliniken Essen  Medzinische Fakultal der Universitat Duisburg- Essen  Ethik-Kommisssion  Robert-Koch-Sir. 9-11 45147 Essen GERMANY |  | |  |
| Justus-Liebig- Universitats Giessen Ethik-Kommission des Fachbereichs Medizin Gaffkystr. 11c  35385 Giessen  GERMANY |  | |  |
| Ethikkornmission der Medizinische Fakultat der O!to-von-Guericke- Universilat Magdeburg Leipziger Sir. 44  39120 Magdeburg  GERMANY |  | |  |
| Medizinische Hochschule Hannover Carl-Neuberg-Str. 1  30625 Hannover  GERMANY |  | |  |
| Ethikkommission der Arztekammer Weslfalen- Lippe und der Med.  Fakul!at der *WNU*  Munster  Von-Esmarch-Str.62  48149 Munster GERMANY |  | |  |
| Universitat Rostock Med. Fakultat St.Georg Str. 108  18055 Rostock  GERMANY |  | |  |
| Landesarztekammer Hessen  Im Vogelgesang 3  60488 Frankfurt/Main  GERMANY |  | |  |
| Landesarz1ekammer Bertin  Ethik-Kommission Friedrichstr. 16  10969 Berlin GERMANY |  | |  |
| Landesarztekammer Hessen  Im Vogelgesang 3  60488 Frankfurt/Main GERMANY |  | |  |
|  | Pirkanmaan Sairaanhoitopiirin  kuntayhtyma Eettisen toimikunta  Teiskontie 35, Tampere PL2000  33521 Tampere  FINLAND | |  |
|  | Pirkanmaan Sairaanhoitopiirin kuntayhtymii Eettisen toimikunta  Teiskonlie 35, Tamoere | |  |
|  | PL 2000  33521 Tampere  FINLAND | |  |
|  | Pirkanmaan Sairaanhoitopiirin kuntayhtyma Eettisentoimikunta Teiskonlie 35, Tampere PL2000  33521 Tampere FINLAND | |  |
|  | Pirkanmaan  Sairaanhoitopiirin lkuntayhtyma Eettisen toimikunta  Teiskontie 35, Tampere PL2000  33521 Tampere  iFINLAND | |  |
|  | Pirkanmaan Sairaanhoitooiirin  kuntayhtyma Eettisen toimlkunta  Teiskontie 35, Tampere PL2000  33521 Tampere FINLAND | |  |
|  | CPP Sud-Est Ill  Porte 16  H6pital Hotel Dieux 1, Place de l'Hopital  69288 LYON **Cedex** 02  FRANCE | |  |
|  | CPP Sud-Est Ill Porte 16  H6pital Hotel Dieux 1, Place de l'H6pital  69288 LYON Cedex 02 FRANCE | |  |
|  | CPP Sud-Est Ill Porte 16  H6oital Hotel Dieux  1, Place de I' Hopital 69288 LYON Cedex 02 FRANCE | |  |
|  | CPP Sud-Est Ill  Porte 16  Hopital Hotel Dieux 1, Place de l'Hopital  69288 LYON Cedex 02 FRANCE | |  |
|  | CPP Sud-Est Ill  Porte 16  H6pital Hotel Dieux 1, Place de l'Hopital  69288 LYON Cedex 02  FRANCE | |  |
|  | CPP Sud-Est Ill Porte 16  H6pital Hotel Dieux 1, Place de l' H6pital  69288 LYON Cedex 02 FRANCE | |  |
|  | CPP Sud-Est Ill Porte 16  H6pital Hotel Dieux 1, Place de 'l H6pital  69288 LYON Cedex 02 FRANCE | |  |
|  | CPP Sud-Est Ill  Porte 16  H6pital Hotel Dieux 1, Place de l'H6pital  69288 LYON Cedex 02 FRANCE | |  |
|  | CPP Sud-Est Ill Porte 16  Hopital Hotel Dieux 1, Place de l'H6pital  69288 LYON Cedex 02  FRANCE | |  |
|  | CPP Sud-Est Ill Porte 16  Hopital Hotel Dieux  1, Place de I'H6pital  69288 LYON Cedex 02 FRANCE | |  |
|  | CPP Sud-Est Ill  Porte 16  H6pital Hotel Dieux 1, Place de l'H6pital  69288 LYON Cedex 02  FRANCE | |  |
|  | CPP Sud-Est Ill Porte 16  H6pital H6te'I Dieux 1, Place de 1'H6pital  69288 LYON Cedex 02 FRANCE | |  |
|  | CPP Sud-Est Ill  Porte 16  H6pital Hote1 Dieux 1, Place de l' H6pital  69288 LYON Cedex 02 FRANCE | |  |
|  | CPP Sud-Est Ill Porte 16  Hopital Hotel Dieux 1, Place de l'H6pital  69288 LYON Cedex 02 FRANCE | |  |
|  | NHS  Laurie House Colyear Street Derby  DE1 1LJ  UNITED KINGDOM | |  |
|  | NHS  Laurie House Colyear Street Derby  DE1 1LJ  UNITED KINGDOM | |  |
|  | NHS  Laurie House Colyear Street  Derby DE11LJ  UNITED KINGDOM | |  |
|  | NHS  Laurie House Colyear Street Derby DE11LJ  UNITED KINGDOM | |  |
|  | NHS  Laurie House Colyear Street Derby DE11LJ  UNITED KINGDOM | |  |
|  | NHS  Laurie House Colye,ar Street Derby  DE11LJ  UNITED KINGDOM | |  |
|  | NHS  Laurie House Colyear Street Derby  DE11 LJ  UNITED KINGDOM | |  |
|  | NHS  Laurie House Colyear Street Derby DE11LJ  UNITEDKINGDOM | |  |
|  | NHS  Laurie House Colyear Street Derby  DE1 1LJ UNITEDKINGDOM | |  |
|  | NHS  Laurie House Colvear Street  Derby DE11LJ  UNITED KINGDOM | |  |
|  | NHS  Laurie House Colyear Street Derby DE11LJ  UNITED KINGDOM | |  |
| Comitato Etioo per la Sperimentazione Clinica dei Medtcinali  Azienda Ospedaliero- Universitaria Careggi Largo Palagi 1 c/o C.T.O.  50139 Firenze ITALY | Comitato Elic o dell'IRCCS Fondazione  S. Raffaele Del Monte Tabor Di Milano  Via Olgettina, 60  20132 Milano ITALY | |  |
| Comitato Etioo lndipente  di Riferimento individuate dala Regione  Piemonte  *cfo* Assessorata alla Sanita  Corso Regina Margherita 153/bis  10122 Torino ITALY | Comitato Etico dell'IRCCS Fondazione  S. Raffaele Del Monte  Tabor Di Milano Via Olgettina, 60  20132 Milano ITALY | |  |
| ComitatoEtico  dell'IRCCS Fondazione  S. Raffaele Del Monte Tabor Di Milano  Via Olgettina, 60  20132 Milano ITALY | ComitatoEtico  dell'IRCCS Fondazione  S. Raffaele Del Monte Tabor Di Milano  Via Olgettina, 60  20132 Milano ITALY | |  |
| Comitato Etico dell'  IRCCS Fondazione ' lstituto Neurologico Casimiro Mondino' Via Mondino, 2  27100 Pavia ITALY | ComitatoElico  dell'IRCCS Fondazione  S. Raffaele Del Monte Tabor Di Milano  Via Olgettina, 60  20132 Milano ITALY | |  |
| Cornitato Etico per la Sperirnentazione dell'Azienda Ospedaliera Di Padova  Via Giustiniani1 35128 Padova ITALY | Cornitalo Etico dell'IRCCS Fondazione  S. Raffaele Del Monte Tabor Di Milano  Via Olgettina, 60  20132 Milano ITALY | |  |
| CornitatoEtico  dell'Azienda Ospedaliera  S. Carnillo- ForlaniniCirconvallazione Gianicolense,87  00152 Roma ITALY | Cornitato Etico  dell'IRCCS Fondazione  S. Raffaele Del Monte Tabor Di Milano  Via Olgettina, 60  20132 Milano  ITALY | |  |
| Cornitato Etico dell'  Azienda Ospedaliera  S. Antonio Abate di Gallarate  Largo A. Bolio 2  21013 Gallarate (VA) ITALY | Cornitato Etico  dell'IRCCS Fondazione  S. Raffaele Del Monte Tabor Di Milano  Via Olgettina, 60  20132 Milano ITALY | |  |
| Comitato Etico lndipendente Locale Azienda Ospedaliera ' Ospedale Policlinico Consorziale" di Bari P.zza G.Cesare, 11  70124 Bari  ITALY | Comita to Etico dell'IRCCSFondazione  S. Raffaele Del Monte TaborDi Milano  Via Olgetlina, 60  20132 Milano ITALY | |  |
| Comitato Etico dell'Azienda Policlinioo Umberto I di Roma Viale del Policlinico 155 00161 Roma  ITALY | Comitato Etico delrlRCCS Fondazione  S. Raffaele Del Monte Tabor Di Milano  Via Olgettina, 60  20132 Milano  ITALY | |  |
| Comitato Etico Unico per la Provincia di Parma Via A. gramsci, 14 43126 Parma  ITALY | Comitato Etico dell'IRCCS Fondazione  S. Raffaele Del Monte Tabor Di Milano  Via Olgettlna, 60  20132 Milano  ITALY | |  |
|  | KomsJa Bioetyki Uniwersy1etu Medycznego **W** l odzi AL. Kosciuszki 4  90-419 lodz  POLAND | |  |
|  | Komsja Bioetyki Uniwersy1etu Medycznego **W** l odzi AL. Kosciuszki 4  90-419 l6dz POLAND | |  |
|  | Komsja Bioetyki  Uniwersytetu Medycznego **W** l odzi AL. Kosciuszki 4  90-419 l6di:  POLAND | |  |
|  | Komsja Bioetyki Uniwersytetu  Medycznego W Lodz i AL. Kosciuszki 4  90-419 Lodz  POLAND | |  |
|  | Komsja Bioetyki Uniwersytetu Medycznego W Lodzi AL. Kosciuszki 4  90-419 Lodz POLAND | |  |
|  | Komsja Bioetyki  Uniwersytetu Medycznego W l odzi AL. Kosciuszki 4  90-419Lodz POLAIND | |  |
|  | Komsja Bioetyki Uniwersytetu Medycznego W l odzi AL. Kosciuszki 4  90-419Lodz  POLAND | |  |
|  | Komsja Bioetyki Uniwersytetu Medyczne go **W** Lodzi AL. Kosciuszki **4**  90-419 Lodi: POLAND | |  |
|  | KomsJa Bioetyki Uniwersytetu Medycznego **W** l odzi AL. Kosciuszki 4  90-419 lodz  POLAND | |  |
| Ethics Committee of the Military-Medical Academy n.a. **S.M.** irov 6, Lebedeva sir.  St -Petersburg, 1904 RUSSIA | Ethics Committee of Federal Body For Quality Control of Medicines  8, bid. 3, Petrovsky  bulvar  Moscow, 127015 RUSSIA | |  |
| Ethics Committee of St.-  Petersburg State Medical University n.a. acad. LP.Pavlov  10, Rentgena sir.  St -Petersburg, 197101 RUSSIA | Ethics Committee of  Federal Body For Quality Control of Medicines  8, bid. 3, Petrovsky bulvar  Moscow, 127051 RUSSIA | |  |
| Ethics Committee of  Russian State Medical University  1, Ostrovityanova str.  Moscow, 117997 RUSSIA | Ethics Committee of  Federal Body For Quality Control of Medicines  8, bid. 3, Petrovsky bulvar  Moscow, 127051 RUSSIA | |  |
| Local Ethics Committee  NO MF MSCh #1 AMO  Zlil  26, Bakinskaya sir.  Moscow, 115516 RUSSIA | Ethics Committee of  Federal Body For Quality Control of Medicines  8, bid. 3, Petrovsky bulvar  Moscow, 127051  RUSSIA | |  |
| Ethics Committee of City Clinical Hospital #n/6, Dvintsev sir. Moscow, 127018 RUSSIA | Ethics Committee of Federal Body For Quality Control of Medicines  8, bid. 3, Petrovsky bulvar  Moscow, 127051 RUSSIA | |  |
| Ethics Committee of the  Instituteof the Human Brain of the Russian Academy of Sciences 9, akad. Pavlova sir. St. Petersburg, 197376  RUSSIA | Ethics Committee of  Federal Body For Quality Control of Medicines  8, bid. 3, Petrovsky bulvar  Moscow, 127051  RUSSIA | |  |
| Ethics Committee at the 'City Clinical Hospital No. 33' 54, Lenina avenue  Nizhny Novgoor d, 603076  RUSSIA | Ethics Committee of Federal Body For Quality Control of Medicines  8, bid. 3, Petrovsky bulvar  Moscow, 127051  RUSSIA | |  |
| Ethics Committee of  Federal State Institution ' Siberian district Medical Center of the Federal Medical-Biological Agency of Russia"  13, Kainskaya sir.  Novosibirsk, 630007 RUSSIA | Ethics Committee of  Federal Body For Quality Control of Medicines  8, bid. 3, Petrovsky bulvar  Moscow, 127051 RUSSIA | |  |
| Ethics Committee of City  Clinical Hospital #1  6, Zalesskogo str.,  Novosibirsk, 630047 RUSSIA | Ethics Committee of Federal Body For Quality Control of Medicines  8, bid. 3, Petrovsky bulvar  Moscow, 127051  RUSSIA | |  |
| Local Ethics Committee GCUHPE "Kazan State Medical University of the Federal Agency on Healthcare and Social  Development"  49, Butlerova str.  Kazan, 420012 RUSSIA | Ethics Committee of Federal Body For Quality Control of Medicines  8, bid. 3, Petrovsky bulvar  Moscow, 127051  RUSSIA | |  |
|  | Regionala  elikprovnlnsniimnden i Stockholm att:Vetenskapllg sekr. Box 289  Karolinska Institute! Nobels vag 12A SWEDEN | |  |
|  | Regionala etlkprovninsnamnden i Stockholm att:Vetenskaplig sekr. Box 289  Karollnska Institute! Nobels viig 12A  SWEDEN | |  |
|  | Regionala etikprovnnisniimndeni  Stockholm att:Vetenskaplig sekr. Box 289 KarolinskaInstitute!  Nobels vag 12A SWEDEN | |  |
| Commission for Ethic Issues of Zaporizhzhya Medical Academy of  PostgraduateEducation 20, Vintera Bulvar  Zaporizhzhya, 69096 UKRAINE | Central Commission for Ethic Issuesof the Ministry of Health of Ukraine  5 Narodnogo Opolchennya Sir. Kyiv, 03068 MCP UKRAINE | |  |
| Ethic Commeetee of Municipal Institution  ' OdesaRegional Clinical Hospital"  26, Zabolotnogo Sir.  ODESA, 65025 UKRAINE | Central Commission for Ethic Issuesof the Ministry of Health of Ukraine  5 Narodnogo  Opolchennya Sir. Kyiv 03068 MCP  UKRAINE | |  |
| Cornission for Ethic Issues of Ukrainian State Scientific-Research Institute of Medical and Social Problems of Disability  1a Radyanskyy prov, Dnipropetrovsk, 49027  UKRAINE | Central Commission for Ethic Issues of the Ministry of Health of Ukraine  5 Narodnogo Opolchennya Str. Kyiv, 03068 MCP UKRAINE | |  |
| Etthic Commeetee of lvano-Frankivsk Region Clinical Hospital  **91,** Fed'kovicha Sir.  lvano-Frankivsk, 76008 UKRAINE | Central Commission for Ethic Issues of the Ministry of Health of Ukraine  5 Narodnogo Opolchennya Str. Kyiv, 03068 **MCP**  UKRAINE | |  |
| Comission for Ethic Issues of State Medical  and Preventive  Treatment Instituiton 'Central Clinical Hospital of Ukrzaliznytsya'  5 Balakirevaprov.  Kharkiv, 61018 UKRAINE | Central Commission for Ethic Issues of the Ministrv of Health of  Ukraine  5 Narodnogo Opolchennya Sir. Kyiv, 03068 MCP UKRAII NE | |  |
| Comiss ion for Ethic  Issues of Vinnytsya Regional Psychoneurological Hospital n.a. acad. 0.1. Yushenko  109 Pyrogova vul.  Vinnytsya, 21005 UKRAINE | Central Commission for  Ethic Issues of the Ministry of Health of Ukraine  5 Narodnogo Opolchennya Sir. Kyiv, 03068 MCP UKRAINE | |  |
| Etihic Commettee of Lviv Region Clinical Hospital  6, Nekrasova Sir.  Lviv, 79010 UKRAINE | Central Commission for Ethic Issues of the Ministry of Health of Ukraine  5 Narodnogo Ooolchennva Sir.  Kyiv, 03068 MCP UKRAINE | |  |
| Ethic Commettee of | Central Commission for | |  |
| Region Clinical Centre of | Ethic Issues of the | |  |
| Neurology and | Ministry of Health of | |  |
| Neurosurgery in | Ukraine | |  |
| Uzhgorod | 5 Narodnogo | |  |
| 24, v. Peremogy | Opolchennya Sir. | |  |
| Uzhgorod, 88018 | Kyiv, 03068 MCP | |  |
| UKRAINE | UKRAINE | |  |
| Commission for Ethic | Central Commission for | |  |
| Issuesof Municipal | Ethic i'ssues of the | |  |
| Institution of Health Care | Ministry of Health of | |  |
| 'City Clinical Hospital | Ukraine | |  |
| No.7' | 5 Narodnogo | |  |
| 266 Saltivske shose, | Opolchennya Sir. | |  |
| Kharkiv, 61178 | Kyiv, 03068 MCP | |  |
| UKRAINE | UKRAINE | |  |
|  | Regional komite for medidinsk cg helseafglig forskninosetikk  S0r-0st-C  Postboks 1130 Blindem  0318 Oslo NORWAY | |  |
|  | Regional komite for  medidinskog helsefaglig forskningsetikk  S0r-0st-C  Postboks 1130 Blindern  0318 Oslo NORWAY | |  |
|  | Regional komite for  medidinsk og helsefaglig forskningsetikk  S0r-0st-C  Postboks 1130 Blindern  0318 Oslo NORWAY | |  |
|  | Regional komite for  medidinskog helsefaglig forskninqsetikk  S0r-0st -C  Postboks 1130 Blindem  0318 Oslo NORWAY | |  |
| Camile de Elica Hospital Clinico  Universidad de Chile Santos Dumont 999 4° piso Sector D Santiago  CHILE |  | |  |
| Camile Etico Cientifico Servicio de Salud MetropoiltanoOriente Salvador 364 Providencia Santiago CHILE |  | |  |
| Comite Etico Cientifico Servicio de Salud  Metropolitano Sur Santa Rosa 3453  Santiago CHILE |  | |  |
| Comite Etico Cientifico  Servicio de Salud Metropoltiano Oriente Salvador 364 Santiago  CHILE |  | |  |
| Comite Etico Cientifico Servicio del Salud Vina del Mar-Quillota Hospital Dr. Gustavo Fricke  Alvarez 1532, 2°Piso,  Oficina 3 Vina del Mar CHILE |  | |  |
| Ethics Committee  Medical Faculty and Teaching Hospital OLOMOUC  I.P. Pavlova 6  775 20 Olomouc  Czech Reoublic | Ethics Committee  Medical Faculty and Teaching Hospital  OLOMOUC  I.P. Pavlova 6  775 20 Olomouc Czech Republic | |  |
|  | Comissao de Etica para a lnvesligai;:ao Clinica (CEIC)  Parque de Sai.ide de Lisboa  Avenida do Brasil, 53 -  Pav. 17-A  1749-004 Lisboa PORTUGAL | |  |
|  | Comissao de Etica para a lnvesligai;:ao Clinica (CEIC)  Parque de Saude de Lisboa  Avenida do Brasil, 53 -  Pav. 17-A  1749-004 Lisboa PORTUGAL | |  |
|  | Comissao de Eticapara a lnvestigai;:ao Cllnica (CEIC)  Parqlle de Saudede Lisboa  Avenidado Brasil, 53 - Pav. 17-A  1749-004 Lisboa PORTUGAL | |  |
|  | Stegmetc  Louis Armstmngweg 78  1311 RL Almere  THE NETHERLANDS | |  |
|  | Stegmetc  Louis Armst rongweg78 1311 RLAlmere  THE NETHERLANDS | |  |
|  | Stegmetc | |  |
|  | Louis Armstrongweg 78 | |  |
|  | 1311 IRL Almere | |  |
|  | THE NETHERLANDS | |  |
|  | Stegmetc  Louis Armstrongweg 78  1311 RL Almere  THE NETHERLANDS | |  |
|  | De Videnskabseliske Komiteer i Region Hovedstaden KongensVamge 2  3400 Hillernd DENMARK | |  |
|  | De Videnskabseliske  Komiteeri Region Hovedstaden Kongens Vamge 2  3400 Hillernd DENMARK | |  |
|  | De Videnskabsetiske Komiteer i Region Hovedstadne KongensVrenge 2  3400 Hillernd  DENMARK | |  |
| Republic of Turkey | Republic of Turkey | |  |
| IstanbuI University | (T.R.) | |  |
| Cerrahpa9a Medical  Faculty | Ministry of Health  Drug and Pharmacy | |  |
| 34303 Cerrahpa a *I* | General Directorate | |  |
| istanbul | <;ankiri Cad. No: 57 | |  |
| TURKEY | Oi9kapi | |  |
|  | Ulus 06060 Ankara | |  |
|  | TURKEY | |  |
| Hacettepe University | Republic of Turkey | |  |
| Medical Faculty | (T.R.) | |  |
| medical, Surgery and | Ministry of Health | |  |
| Drug Research Ethics | Drug and Pharmacy | |  |
| Committee | General Directorate | |  |
| 06100 S1hh1ye /Ankara | <;ank iri Cad. No: 57 | |  |
| TURKEY | Oi9kapi | |  |
|  | Ulus 06060 Ankara | |  |
|  | TURKEY | |  |
| Gazi University Medical Faculty  Local Ethics Committee 06500 Be evler / Ankara TURKEY | Republic of Turkey  /T.R.l  Ministry of Health Drug and Pharmacy General Directorate Canklri Cad. No: 57 Di kapi  Ulus 06060 Ankara TURKEY | |  |
| Dokuz Eyliil University  Medical Faculty Pharmaceutical Research Local Ethics Committee  35340 lnciralt1 / Izmir TURKEY | Republic of Turkey  (T.R.)  Ministry of Health Drug and Pharmacy General Directorate Canklri Cad. No: 57 Di kapi  Ulus 06060 Ankara  TURKEY | |  |
| Uludag University Medical Faculty  Local Ethics Committee Goriikle Kampiisii 16059 Bursa  TURKEY | Republic of Turkey (T.R.)  Ministry of Health Drug and Pharmacy  General Directorate  <;ankiri Cad. No: 57 Di kapi  Ulus 06060 Ankara  TURKEY | |  |
| Kocaeli University Human Researches Ethics Committee Umuttepe Yerleskesi 41380 Umuttepe/lZMIT TURKEY | Republic of Turkey (T.R.)  Ministry of Health Drug and Pharmacy General Directorate  !;ank iri Cad. No: 57 Di kapi  Ulus 06060 Ankara  TURKEY | |  |
| Izmir Education and Research Hospital Saim <;1kriki;:1 Street No 59  Bozyaka-lZMIR  TURKEY | Republic of Turkey (T.R.)  Ministry of Health Drug and Pharmacy  General Directorate  <;ankiri Cad. No: 57 Di kapi  Ulus 06060 Ankara  TURKEY | |  |
| Tepecik Education and | Republic of Turkey | |  |
| Research Hospital | (T.R.) | |  |
| Local Ethics Committee | Ministry of Health | |  |
| Yenisehir 35310-IZMIR | Drug and Pharmacy | |  |
| TURKEY | General Directorate | |  |
|  | Cankiri Cad. No: 57 | |  |
|  | Di kapi | |  |
|  | Ulus 06060 Ankara | |  |
|  | TURKEY | |  |

**C. TOWER.**

| **INSTITUTIONAL REVIEW BOARD (IRB)/INDEPENDENT ETHICS COMMITTEE (IEC) Name and Address** | |  |
| --- | --- | --- |
| **Local IRB/IEC** | **National IRB/IEC** |  |
| Hunter New England Human Research Ethics Committee  Lookout Road  New Lambton Heights 2305  New South Wales AUSTRALIA |  |  |
| Hunter New England Human Research Ethics Committee  Lookout Road  New Lambton Heights 2305  New South Wales AUSTRALIA |  |  |
| St. Vincents Hospital Human Research Ethics Committee  41 VICTORIA PARADE  Fitzroy 3065  Victoria AUSTRALIA |  |  |
| Geelong Hospital EC Barwon Health Research and Ethics Advisory Committee The Geelong Hospital PO Box 281  Geelong 3220  Victoria AUSTRALIA |  |  |
| Flinders Clinical Research Ethics Committee  Human Research Ethics Room 2A221  Flinders Medical Centre Bedford Park  5042  South Australia AUSTRALIA |  |  |
| Hunter New England Human Research Ethics Committee  Lookout Road  New Lambton Heights 2305  New South Wales AUSTRALIA |  |  |
| Austin & Repatriation Med. Center Gastroenterology and Hepatology  Studley Road Heidleberg 3084  Victoria AUSTRALIA |  |  |
|  | Ethikkommission der Stadt Wien  3., Thomas-Klestil-Platz 8/2  TownTown Wien  1030  AUSTRIA |  |
|  | UZ Leuven UZ Gasthuisberg Herestraat 49 Leuven  3000  BELGIUM |  |
|  | UZ Leuven UZ Gasthuisberg Herestraat 49 Leuven  3000  BELGIUM |  |
|  | UZ Leuven UZ Gasthuisberg Herestraat 49 Leuven  3000  BELGIUM |  |
|  | UZ Leuven UZ Gasthuisberg Herestraat 49 Leuven  3000  BELGIUM |  |
| LEC of Republic scientific practical 24, F. Skoryny Str. Minsk  220114  BELARUS |  |  |
| LEC of City Clinical Hospital 5  9 Filatova str. Minsk 220026  BELARUS |  |  |
| EC "Vitebsk regional clinical hospital" Voinov Ethics Committee of the Institution of Health Care " Vitebsk regional clinical hospital" Voinov Internacionalistov str. 37 Vitebsk  210037  BELARUS |  |  |
| LEC of the City Clinical Hospital # 9  8 Semashko Str., Minsk, 220116, Belarus  Minsk 220116 BELARUS |  |  |
| LEC of the Grodno Regional Hospital 52, Leniskogo  Komsomola boulevard, Grodno  230017  BELARUS |  |  |
| Comite d'ethique a la Recherche du CHA - Hopital de l'Enfant 1401, 18e rue Quebec  G1J 1Z4  Quebec CANADA |  |  |
| Queen's University Health Sciences REB Office of Research Services  Fleming Hall/Jemmett Wing  Kingston K7L 3N6  Ontario CANADA |  |  |
| Saskatchewan Drug Research Institute University Of Saskatchewan Room 5681, Royal University Hospital 103 Hospital Drive Saskatoon  S7N 0W8  Saskatchewan CANADA |  |  |
| Canadian Shield Ethics Review Board  501 Deerhurst Drive,  Suite 102 Burlington L7L 5T1  Ontario CANADA |  |  |
| Horizon Health Network Research Ethics Board 400 University Avenue Saint John  E2L 4L2  New Brunswick CANADA |  | |
| Comite Ethique de la recherche  Hopital Maisonneuve- Rosemont  5415, boul. De L'Assomption Montreal  H1T 2M4  Quebec CANADA |  | |
| Canadian Shield Ethics Review Board  501 Deerhurst Drive,  Suite 102 Burlington L7L 5T1  Ontario CANADA |  | |
| Comite de Etica de la Investigacion S.S.M Central  Victoria subercaseaux 381, 5° piso, Santiago Centro  Santiago 8360160  RM Region Metropolitan CHILE |  | |
| Comite de Etica de la Investigacion S.S.M Central  Victoria subercaseaux 381, 5° piso, Santiago Centro  Santiago 8360160  RM Region Metropolitan CHILE |  | |
| Comite de Etica de la Investigacion S.S.M. Sur Av. Santa Rosa # 3453 Santiago  8360160  RM Region Metropolitan CHILE |  | |
|  | IEC of Beijing Hospital No.1, Dahua Road Beijing  100730  CHINA | |
|  | IEC of Beijing Hospital No.1, Dahua Road Beijing  100730  CHINA | |
|  | IEC of Beijing Hospital No.1, Dahua Road Beijing  100730  CHINA | |
|  | IEC of Beijing Hospital No.1, Dahua Road Beijing  100730  CHINA | |
| IEC of Xuanwu Hospital Capital Medical University  No.45, Changchun Street  Beijing 100053 CHINA | IEC of Beijing Hospital No.1, Dahua Road Beijing  100730  CHINA | |
| Independent Ethics Committee of Beijing Friendship Hospital  IEC of Beijing Friendship Hospital  No.95, Yong'an Road Beijing  100050  CHINA | IEC of Beijing Hospital No.1, Dahua Road Beijing  100730  CHINA | |
| IEC of First Hospital, Jilin University No.71, Xinmin Street Changchun  130021  CHINA | IEC of Beijing Hospital No.1, Dahua Road Beijing  100730  CHINA | |
|  | IEC of Beijing Hospital No.1, Dahua Road Beijing  100730  CHINA | |
| IEC of First Hospital of China Medical University No.155, Najing Street(north)  Shenyang 110001  CHINA | IEC of Beijing Hospital No.1, Dahua Road Beijing  100730  CHINA | |
|  | IEC of Beijing Hospital No.1, Dahua Road Beijing  100730  CHINA | |
|  | IEC of Beijing Hospital No.1, Dahua Road Beijing  100730  CHINA | |
| IEC of Third Affiliated Hospital of Sun Yat-Sen University No.600,Tianhe Road Guangzhou  510630  CHINA | IEC of Beijing Hospital No.1, Dahua Road Beijing  100730  CHINA | |
|  | IEC of Beijing Hospital No.1, Dahua Road Beijing  100730  CHINA | |
|  | IEC of Beijing Hospital No.1, Dahua Road Beijing  100730  CHINA | |
| Independent Ethics Committee of Huashan Hospital of Fudan Un IEC of Huashan Hospital of Fudan University No.12, Wulumuqi Road (Middle)  Shanghai 200040  CHINA | IEC of Beijing Hospital No.1, Dahua Road Beijing  100730  CHINA | |
|  | IEC of Beijing Hospital No.1, Dahua Road Beijing  100730  CHINA | |
| IEC of First Affiliated Hospital of Soochow University  IEC of The First Affiliated Hospital of Soochow University No.188,Shizi Street Suzhou  215006  CHINA | IEC of Beijing Hospital No.1, Dahua Road Beijing  100730  CHINA | |
| IEC of The Affiliated Hosp. of Qingdao University No.16,Jiangsu Road Qingdao  266003  CHINA | IEC of Beijing Hospital No.1, Dahua Road Beijing  100730  CHINA | |
| IEC of Sir Run Run Shaw Hospital No.3,Qingchun Road (East)  Hangzhou 310016  CHINA | IEC of Beijing Hospital No.1, Dahua Road Beijing  100730  CHINA | |
| IEC of Hebei General Hospital  No.348, Heping Road (West)  Shijiazhuang 050051 CHINA | IEC of Beijing Hospital No.1, Dahua Road Beijing  100730  CHINA | |
|  | IEC of Beijing Hospital No.1, Dahua Road Beijing  100730  CHINA | |
| IEC of General Hospital of the Second Artillery Force of PLA  No.16, Xinjiekouwai Street  Beijing 100088 CHINA | IEC of Beijing Hospital No.1, Dahua Road Beijing  100730  CHINA | |
| IEC of The Second Affiliated Hospital of Guangzhou Medical College No.250,Changgang Road (East) Guangzhou  510260  CHINA | IEC of Beijing Hospital No.1, Dahua Road Beijing  100730  CHINA | |
|  | IEC of Beijing Hospital No.1, Dahua Road Beijing  100730  CHINA | |
| First Affiliated Hospital of Shanxi Medical University  No.85, Jiefang Road (South)  030001  CHINA | IEC of Beijing Hospital No.1, Dahua Road Beijing  100730  CHINA | |
|  | IEC of Beijing Hospital No.1, Dahua Road Beijing  100730  CHINA | |
|  | IEC of Beijing Hospital No.1, Dahua Road Beijing  100730  CHINA | |
| IEC of Peking University First Hospital  No 8, Xishiku Street,Xicheng District Beijing  100034  CHINA | IEC of Beijing Hospital No.1, Dahua Road Beijing  100730  CHINA | |
| IEC of Beijing Boai Hospital  No10. Jiaomen North Road,Fengtai District Beijing  100068  CHINA | IEC of Beijing Hospital No.1, Dahua Road Beijing  100730  CHINA | |
| IEC of Second Hospital of Shandong University No.247, Beiyuan Street Jinan  250033  CHINA | IEC of Beijing Hospital No.1, Dahua Road Beijing  100730  CHINA | |
| IEC of DrumTower Hospital of Nanjing University Medical School  No.321, Zhongshan Road  Nanjing 210008  CHINA | IEC of Beijing Hospital No.1, Dahua Road Beijing  100730  CHINA | |
|  | IEC of Beijing Hospital No.1, Dahua Road Beijing  100730  CHINA | |
| Multicentricka eticka komise FN u sv. Anny v Brne  Pekarska 53 Brno  656 91  CZECH REPUBLIC |  | |
| Eticka komise Fakultni nemocnice Ostrava  17. listopadu 1790 Ostrava-Poruba 70852  CZECH REPUBLIC |  | |
| Eticka komise Krajska zdravotni, as - Nemocnice Teplice, oz Eticka komise Krajska zdravotni, as - Nemocnice Teplice, oz Duchcovska 53 Teplice  41529  CZECH REPUBLIC |  | |
| Research Ethics Committee of the University of Tartu Lossi 3-318  Office of Research and Development University of Tartu Tartu 51003  ESTONIA |  | |
| Research Ethics Committee of the University of Tartu Lossi 3-318  Office of Research and Development University of Tartu Tartu  51003  ESTONIA |  | |
|  | CPP Sud-Est 3 CPP Lyon B Hôpital Hôtel Dieu Porte 16  1, place de l'Hôpital Lyon Cedex 02  69288  FRANCE | |
|  | CPP Sud-Est 3 CPP Lyon B Hôpital Hôtel Dieu Porte 16  1, place de l'Hôpital Lyon Cedex 02  69288 FRANCE | |
|  | CPP Sud-Est 3 CPP Lyon B Hôpital Hôtel Dieu Porte 16  1, place de l'Hôpital Lyon Cedex 02  69288  FRANCE | |
|  | CPP Sud-Est 3 CPP Lyon B Hôpital Hôtel Dieu Porte 16  1, place de l'Hôpital Lyon Cedex 02  69288  FRANCE | |
|  | CPP Sud-Est 3 CPP Lyon B Hôpital Hôtel Dieu Porte 16  1, place de l'Hôpital Lyon Cedex 02  69288  FRANCE | |
|  | CPP Sud-Est 3 CPP Lyon B Hôpital Hôtel Dieu Porte 16  1, place de l'Hôpital Lyon Cedex 02  69288  FRANCE | |
|  | CPP Sud-Est 3 CPP Lyon B Hôpital Hôtel Dieu Porte 16  1, place de l'Hôpital Lyon Cedex 02  69288  FRANCE | |
|  | Ethikkommision Universität Erlangen- Nürnberg  Ethik-Kommission der Medizinischen Fakultät Friedrich-Alexander- Krankenhausstraße 12  Erlangen 91054 GERMANY | |
|  | Ethikkommision Universität Erlangen- Nürnberg  Ethik-Kommission der Medizinischen Fakultät Friedrich-Alexander- Universität Erlangen- Nürnberg Krankenhausstraße 12 Erlangen  91054  GERMANY | |
|  | Ethikkommision Universität Erlangen- Nürnberg  Ethik-Kommission der Medizinischen Fakultät Friedrich-Alexander- Universität Erlangen- Nürnberg Krankenhausstraße 12 Erlangen  91054  GERMANY | |
|  | Ethikkommision Universität Erlangen- Nürnberg  Ethik-Kommission der Medizinischen Fakultät Friedrich-Alexander- Universität Erlangen- Nürnberg Krankenhausstraße 12 Erlangen  91054  GERMANY | |
|  | Ethikkommision Universität Erlangen- Nürnberg  Ethik-Kommission der Medizinischen Fakultät Friedrich-Alexander- Universität Erlangen- Nürnberg Krankenhausstraße 12 Erlangen  91054  GERMANY | |
|  | Ethikkommision Universität Erlangen- Nürnberg  Ethik-Kommission der Medizinischen Fakultät Friedrich-Alexander- Universität Erlangen- Nürnberg Krankenhausstraße 12 Erlangen  91054  GERMANY | |
|  | Ethikkommision Universität Erlangen- Nürnberg  Ethik-Kommission der Medizinischen Fakultät Friedrich-Alexander- Universität Erlangen- Nürnberg Krankenhausstraße 12 Erlangen  91054  GERMANY | |
|  | Ethikkommision Universität Erlangen- Nürnberg  Ethik-Kommission der Medizinischen Fakultät Friedrich-Alexander- Universität Erlangen- Nürnberg Krankenhausstraße 12 Erlangen  91054  GERMANY | |
|  | Ethikkommision Universität Erlangen- Nürnberg  Ethik-Kommission der Medizinischen Fakultät Friedrich-Alexander- Universität Erlangen- Nürnberg Krankenhausstraße 12 Erlangen  91054  GERMANY | |
|  | National Ethics Committee  284, Messoghion Avenue Cholargos Athens  15562  GREECE | |
|  | National Ethics Committee  284, Messoghion Avenue Cholargos Athens  15562  GREECE | |
| CEI Hospital General de México "Dr. Eduardo Liceaga"  Av. Dr. Balmis 148 Col. Doctores  6726  México, D.F. MEXICO |  | |
| CEI de la Facultad de Medicina y HU de la UANL  Av. Madero y Gonzalitos sn  Col. Centro Monterrey 64460  Nuevo Leon MEXICO |  | |
|  | Catharina Ziekenhuis METC  Michelangelolaan 2 Eindhoven  5623 EJ NETHERLANDS | |
|  | Catharina Ziekenhuis METC  Michelangelolaan 2 Eindhoven  5623 EJ NETHERLANDS | |
|  | Catharina Ziekenhuis METC  Michelangelolaan 2 Eindhoven  5623 EJ NETHERLANDS | |
|  | Catharina Ziekenhuis METC  Michelangelolaan 2 Eindhoven  5623 EJ NETHERLANDS | |
|  | Catharina Ziekenhuis METC  Michelangelolaan 2 Eindhoven  5623 EJ NETHERLANDS | |
| Metropolitan Medical Center Institutional Review Board  1357 G. Masangkay St. Tondo, Manila PHILIPPINES |  | |
| Makati Medical Center Institutional Review Board  No. 2 Amorsolo St. Legaspi Village, Makati City  1229  PHILIPPINES |  | |
| St. Luke's Medical Center Instiutional Ethics Review Board  Research and Biotechnology Division Main Building, St. Luke's Medical Center  279 E. Rodriguez Sr. Blvd.  Quezon City 1102 PHILIPPINES |  | |
| Cebu Doctors University Hospital Research Ethics Committee CDUH, Gov. M. Roa St.  corner Don Jose Avila St.  Ground Floor, Administrative Bldg Cebu  6000  PHILIPPINES |  | |
|  | Komisja Bioetyczna przy Wojskowej Izbie Lekarskiej  ul. Koszykowa 78 Warszawa  00-909  Mazowieckie POLAND | |
|  | Komisja Bioetyczna przy Wojskowej Izbie Lekarskiej  ul. Koszykowa 78 Warszawa  00-909  Mazowieckie POLAND | |
|  | Komisja Bioetyczna przy Wojskowej Izbie Lekarskiej  ul. Koszykowa 78 Warszawa  00-909  Mazowieckie POLAND | |
|  | Komisja Bioetyczna przy Wojskowej Izbie Lekarskiej  ul. Koszykowa 78 Warszawa  00-909  Mazowieckie POLAND | |
|  | Komisja Bioetyczna przy Wojskowej Izbie Lekarskiej  ul. Koszykowa 78 Warszawa  00-909  Mazowieckie POLAND | |
|  | National Ethics Committee for Clinical Trial of the Medicine 48, Av. Sanatescu Stefan str., Sector 1 Bucharest  011478  ROMANIA | |
|  | National Ethics Committee for Clinical Trial of the Medicine 48, Av. Sanatescu Stefan str., Sector 1 Bucharest  011478  ROMANIA | |
|  | National Ethics Committee for Clinical Trial of the Medicine 48, Av. Sanatescu Stefan str., Sector 1 Bucharest  011478  ROMANIA | |
|  | National Ethics Committee for Clinical Trial of the Medicine 48, Av. Sanatescu Stefan str., Sector 1 Bucharest  011478  ROMANIA | |
| Eticka komisia Jesseniovej lekarskej fakulty UK v Martine Sklabinska 26  Martin 03645 SLOVAKIA | Eticka komisia Martinskej fakultnej nemocnice Kollarova 2  Martin 03659 SLOVAKIA | |
| Eticka komisia Univerzitnej Nemocnice Bratislava  Nemocnica akad. L. Derera  Limbova 5 Bratislava, Kramare 83305  SLOVAKIA | Eticka komisia Martinskej fakultnej nemocnice Kollarova 2  Martin 03659 SLOVAKIA | |
| Eticka komisia Univerzitnej nemocnice Bratislava  Nemocnica Ruzinov Ruzinovska 6 Bratislava  82606  SLOVAKIA | Eticka komisia Martinskej fakultnej nemocnice Kollarova 2  Martin 03659 SLOVAKIA | |
| Fakultna nemocnica s poliklinikou J.A.Remaina Presov  Eticka komisia Holleho 14  Presov 081 81  Slovak Republic SLOVAKIA | Eticka komisia Martinskej fakultnej nemocnice Kollarova 2  Martin 03659 SLOVAKIA | |
|  | CEIC Hospital Vall d'Hebron  Passeig de la Vall d'Hebron, 119-129 Barcelona  08035  Barcelona SPAIN | |
|  | CEIC Hospital Vall d'Hebron  Passeig de la Vall d'Hebron, 119-129 Barcelona  08035  Barcelona SPAIN | |
|  | CEIC Hospital Vall d'Hebron  Passeig de la Vall d'Hebron, 119-129 Barcelona  08035  Barcelona SPAIN | |
|  | CEIC Hospital Vall d'Hebron  Passeig de la Vall d'Hebron, 119-129 Barcelona  08035  Barcelona SPAIN | |
|  | CEIC Hospital Vall d'Hebron  Passeig de la Vall d'Hebron, 119-129 Barcelona  08035  Barcelona SPAIN | |
|  | Ethics Committee Karolinska Institute, Registrator  Nobels väg 5 Do Not Use 171 77 SWEDEN | |
|  | Ethics Committee Karolinska Institute, Registrator  Nobels väg 5 Do Not Use 171 77  SWEDEN | |
|  | Ethics Committee Karolinska Institute, Registrator  Nobels väg 5 Do Not Use 171 77  SWEDEN | |
| Institutional Review Board, Faculty of Medicine, Chula. U. Faculty of Medicine, Chulalongkorn University  1873 Rama 4 Road, Patumwan, Bangkok  10330  THAILAND |  | |
| Siriraj Institutional Review Board Siriraj Institutional Review Board  2 Wanglang Road, Bangkoknoi Bangkok  10700  THAILAND |  | |
| Local Ethic committee of national Institute of Neurology.  Rue Jébal Lakhdhar La Rabta Bab Saadoun 1007 Tunis  TUNISIA |  | |
| Hôpital Charles Nicolle Boulevard 9 Avril Tunis  TUNISIA |  | |
| Local Ethic committee of Razi hospital.  Rue des orangers – La  Manouba 2010, TUNISIA |  | |
| Hôpital Habib Bourguiba Service De Carcinologie Mé  Avenue El Ferdaous Sfax  3029  TUNISIA |  | |
| Istanbul Universitesi Tip Fakultesi Yerel Etik Kurulu  Istanbul Universitesi Istanbul Tip Fakultesi Tıbbi Arastirmalar Yerel Etik Kurulu  Capa Istanbul 34000 TURKEY |  | |
| Kocaeli University School of Medicine Ethics Committee Kocaeli University School of Medicine Dean's Office Umuttepe  41380  KOCAELI TURKEY | Turkish Drug and Medical Device Agency Söğütözü Mahallesi 2176.Sokak No:5 PK 06520 Çankaya/Ankara TURKEY | |
| Kocaeli University School of Medicine Ethics Committee Kocaeli University School of Medicine Dean's Office Umuttepe  41380  KOCAELI TURKEY | Turkish Drug and Medical Device Agency Söğütözü Mahallesi 2176.Sokak No:5 PK 06520 Çankaya/Ankara TURKEY | |
| Kocaeli University School of Medicine Ethics Committee Kocaeli University School of Medicine Dean's Office Umuttepe  41380  KOCAELI TURKEY | Turkish Drug and Medical Device Agency Söğütözü Mahallesi 2176.Sokak No:5 PK 06520 Çankaya/Ankara TURKEY | |
| Kocaeli University School of Medicine Ethics Committee Kocaeli University School of Medicine Dean's Office Umuttepe  41380  KOCAELI TURKEY | Turkish Drug and Medical Device Agency Söğütözü Mahallesi 2176.Sokak No:5 PK 06520 Çankaya/Ankara TURKEY | |
| Kocaeli University School of Medicine Ethics Committee Kocaeli University School of Medicine Dean's Office Umuttepe  41380  KOCAELI TURKEY | Turkish Drug and Medical Device Agency Söğütözü Mahallesi 2176.Sokak No:5 PK 06520 Çankaya/Ankara TURKEY | |
| Kocaeli University School of Medicine Ethics Committee Kocaeli University School of Medicine Dean's Office Umuttepe  41380  KOCAELI TURKEY | Turkish Drug and Medical Device Agency Söğütözü Mahallesi 2176.Sokak No:5 PK 06520 Çankaya/Ankara TURKEY | |
| Kocaeli University School of Medicine Ethics Committee Kocaeli University School of Medicine Dean's Office Umuttepe  41380  KOCAELI TURKEY | Turkish Drug and Medical Device Agency Söğütözü Mahallesi 2176.Sokak No:5 PK 06520 Çankaya/Ankara TURKEY | |
| Kocaeli University School of Medicine Ethics Committee Kocaeli University School of Medicine Dean's Office Umuttepe  41380  KOCAELI TURKEY | Turkish Drug and Medical Device Agency Söğütözü Mahallesi 2176.Sokak No:5 PK 06520 Çankaya/Ankara TURKEY | |
| Kocaeli University School of Medicine Ethics Committee Kocaeli University School of Medicine Dean's Office Umuttepe  41380  KOCAELI TURKEY | Turkish Drug and Medical Device Agency Söğütözü Mahallesi 2176.Sokak No:5 PK 06520 Çankaya/Ankara TURKEY | |
| Kocaeli University School of Medicine Ethics Committee Kocaeli University School of Medicine Dean's Office Umuttepe  41380  KOCAELI TURKEY | Turkish Drug and Medical Device Agency Söğütözü Mahallesi 2176.Sokak No:5 PK 06520 Çankaya/Ankara TURKEY | |
| Local Ethics Commission of Chernihiv Regional Hospital  25, Volkovich Str Chernihiv  14029  UKRAINE |  | |
| LEC of the "Central clinical hospital of Ukrzaliznytsi"  5, Balakireva pr. Kharkiv  61018  UKRAINE |  | |
| EC for Issues of ME at USSRI of Problems of Disability  1-a, Radyanskiy bst Dnipropetrovsk 49027  UKRAINE |  | |
| EC for Ethics Issues at "Zaporizhzhia Regional Hospital"  10, Orekhovskoe Shosse Zaporizhzhia 69600  UKRAINE |  | |
| EC of Vinnytsya Reg Psychoneuro Hosp n.a. acad. O.I.Yushenko 109, Pirogov Str Vinnitsa  21005  UKRAINE |  | |
| C for Bioethic Issues at Donetsk Nat Med Univ  n.a. M. Gorkiy 16, Ilicha Ave Donetsk 83003 UKRAINE |  | |
| Ethics Commission of Kyiv City Clinical Hospital #4  17, Solomyanska Str. Kyiv  3110  UKRAINE |  | |
| EC at Inst of Neurology Psychiatry and Narcology AMS Ukraine 46, Acad. Pavlov Str Kharkov  61068  UKRAINE |  | |
| Local IEC of Volyn Regional Clinical Hospital  21, Hrushevskyy str. Lutsk  43005  UKRAINE |  | |
| Local IEC of Lviv Regional Clinical Hospital  6, Nekrasova Str.  Lviv 79010 UKRAINE |  | |
| LEC of Ministry of Internal Affairs of Ukraine  19, Manuilskogo Str. Kyiv  4050  UKRAINE |  | |
| Ethics Commission of Kyiv City Clinical Hospital #9  1, Ryzska Str. Kyiv  4112  UKRAINE |  | |
| C for Bioethic Issues at Donetsk Nat Med Univ  n.a. M. Gorkiy 16, Ilicha Ave Donetsk 83003  UKRAINE |  | |
| Committee for Ethics Issues at City Clinical Hospital 6  34, Stalevarov Str.  Zaporizhzhya 69035 UKRAINE |  | |
| LEC of the Ivano- Frankivsk Regional Clinical Hospital  91, Fedkovycha Str., Ivano-Frankivsk 76018  UKRAINE |  | |
| LEC of Poltava Regional Clinical Hospital n.a.  Sklyfosofsky  23 Shevchenko Str. Poltava  36011  UKRAINE |  | |
| LEC of Lviv Regional Clinical Hospital  7 Chernihivska str. Lviv  79010  UKRAINE |  | |
| EC for Ethics Issues at City Clinical Hospital #2 6, Bryulova Str.  Zaporizhzhya 69068 UKRAINE |  | |
| LEC of Lutsk City Clinical Hospital  13, Vidrodzennia str. Lutsk  43024  UKRAINE |  | |
|  | Charing Cross Hospital West London  Room 4W/12, 4th Floor London  W6 8RF  Greater London UNITED KINGDOM | |
|  | Hammersmith, Queen Charlotte's and Chelsea Ethics Committee Hammersmith and Chelsea  Greater London UNITED KINGDOM | |
|  | Charing Cross Hospital West London  Room 4W/12, 4th Floor London  W6 8RF  Greater London UNITED KINGDOM | |
|  | Hammersmith, Queen Charlotte's and Chelsea Ethics Committee Hammersmith and Chelsea  Greater London UNITED KINGDOM | |
|  | Charing Cross Hospital West London  Room 4W/12, 4th Floor London  W6 8RF  Greater London UNITED KINGDOM | |
|  | Hammersmith, Queen Charlotte's and Chelsea Ethics Committee Hammersmith and Chelsea  Greater London UNITED KINGDOM | |
|  | Charing Cross Hospital West London  Room 4W/12, 4th Floor London  W6 8RF Greater London UNITED KINGDOM | |
|  | Hammersmith, Queen Charlotte's and Chelsea Ethics Committee Hammersmith and Chelsea  Greater London UNITED KINGDOM | |
|  | Charing Cross Hospital West London  Room 4W/12, 4th Floor London  W6 8RF  Greater London UNITED KINGDOM | |
|  | Hammersmith, Queen Charlotte's and Chelsea Ethics Committee Hammersmith and Chelsea  Greater London UNITED KINGDOM | |
| Schulman Associates Institutional Reveiw Board  4445 Lake Forest Drive Suite 300  Cincinnati 45242  Ohio  UNITED STATES |  | |
| Schulman Associates Institutional Reveiw Board  4445 Lake Forest Drive Suite 300  Cincinnati 45242  Ohio  UNITED STATES |  | |
| Loma Linda University Adventist Health Institutional Review Board  11188 Anderson Street Loma Linda, CA 92350 UNITED STATES |  | |
| Schulman Associates Institutional Reveiw Board  4445 Lake Forest Drive Suite 300  Cincinnati 45242  Ohio  UNITED STATES |  | |
| Schulman Associates Institutional Reveiw Board  4445 Lake Forest Drive Suite 300  Cincinnati 45242 Ohio  UNITED STATES |  | |
| Schulman Associates Institutional Reveiw Board  4445 Lake Forest Drive Suite 300  Cincinnati 45242 Ohio  UNITED STATES |  | |
| Schulman Associates Institutional Reveiw Board  4445 Lake Forest Drive Suite 300  Cincinnati 45242  Ohio  UNITED STATES |  | |
| Mercy Medical Center- Des Moines Institutional Review Committee  1111 6th Avenue  Des Moines, IA 50314 UNITED STATES |  | |
| Schulman Associates Institutional Reveiw Board  4445 Lake Forest Drive Suite 300  Cincinnati 45242  Ohio  UNITED STATES |  | |
| IRB/OSA  1015 Chestnut Street  Suite 1100  Philadelphia, PA 19107 UNITED STATES |  | |
| Providence Institutional Review Board  5251 NE Glisan Street, Building A, 3rd Floor Portland, OR 97213  UNITED STATES |  | |
| Schulman Associates Institutional Reveiw Board  4445 Lake Forest Drive Suite 300  Cincinnati 45242Ohio  UNITED STATES |  | |
| Schulman Associates Institutional Reveiw Board  4445 Lake Forest Drive Suite 300  Cincinnati 45242 Ohio  UNITED STATES |  | |
| Schulman Associates Institutional Reveiw Board  4445 Lake Forest Drive Suite 300  Cincinnati 45242 Ohio  UNITED STATES |  | |
| Schulman Associates Institutional Reveiw Board  4445 Lake Forest Drive Suite 300  Cincinnati 45242 Ohio  UNITED STATES |  | |
| Schulman Associates Institutional Reveiw Board  4445 Lake Forest Drive Suite 300  Cincinnati 45242  Ohio  UNITED STATES |  | |
| Vanderbilt University Institutional Review Board  504 Oxford House  Nashville, TN 37232 UNITED STATES |  | |
| Schulman Associates Institutional Reveiw Board  4445 Lake Forest Drive Suite 300  Cincinnati 45242  Ohio  UNITED STATES |  | |
| Schulman Associates Institutional Reveiw Board  4445 Lake Forest Drive Suite 300 Cincinnati 45242Ohio  UNITED STATES |  | |
| Institutional Review Board for the Protection of Human Subjects SUNY Upstate Medical University  Weiskotton Hall, Room 1254  750 East Adams Street Syracuse, NY 13210  UNITED STATES |  | |
| Schulman Associates Institutional Reveiw Board  4445 Lake Forest Drive Suite 300  Cincinnati 45242 Ohio  UNITED STATES |  | |
| Schulman Associates Institutional Reveiw Board  4445 Lake Forest Drive Suite 300  Cincinnati 45242 Ohio  UNITED STATES |  | |
| Schulman Associates Institutional Reveiw Board  4445 Lake Forest Drive Suite 300  Cincinnati 45242  Ohio  UNITED STATES |  | |
| Schulman Associates Institutional Reveiw Board  4445 Lake Forest Drive Suite 300  Cincinnati 45242 Ohio  UNITED STATES |  | |
| Schulman Associates Institutional Reveiw Board  4445 Lake Forest Drive Suite 300  Cincinnati 45242 Ohio  UNITED STATES |  | |
| Schulman Associates Institutional Reveiw Board  4445 Lake Forest Drive Suite 300  Cincinnati 45242  Ohio  UNITED STATES |  | |
| Schulman Associates Institutional Reveiw Board  4445 Lake Forest Drive Suite 300  Cincinnati 45242 Ohio  UNITED STATES |  | |
| Western Institutional Review Board  3535 Seventh Avenue, SW  Olympia, WA 98502-  5010  UNITED STATES |  | |
| Schulman Associates Institutional Reveiw Board  4445 Lake Forest Drive Suite 300  Cincinnati 45242  Ohio  UNITED STATES |  | |
| Saint Mary's Health Care  Institutional Review Board  200 Jefferson SE  Grand Rapids, MI 49503 UNITED STATES |  | |
| Research Subjects Protection Programs Institutional Review Board  University of Minnesota D-528 Mayo Building 420 Delaware Street SE Minneapolis, MN 55455 UNITED STATES |  | |
| Schulman Associates Institutional Reveiw Board  4445 Lake Forest Drive Suite 300  Cincinnati 45242  Ohio  UNITED STATES |  | |
| North Mississippi Health Services IRB  830 S Gloster St. Tupelo  38801  Mississippi UNITED STATES |  | |
| Schulman Associates Institutional Reveiw Board  4445 Lake Forest Drive Suite 300  Cincinnati 45242  Ohio  UNITED STATES |  | |
| Schulman Associates Institutional Reveiw Board  4445 Lake Forest Drive Suite 300  Cincinnati 45242 Ohio  UNITED STATES |  | |
| Schulman Associates Institutional Reveiw Board  4445 Lake Forest Drive Suite 300  Cincinnati 45242 Ohio  UNITED STATES |  | |
| Schulman Associates Institutional Reveiw Board  4445 Lake Forest Drive Suite 300  Cincinnati 45242  Ohio  UNITED STATES |  | |
| Western IRB  3535 Seventh Ave. SW Olympia  98502  Washington UNITED STATES |  | |
| Schulman Associates Institutional Reveiw Board  4445 Lake Forest Drive Suite 300  Cincinnati 45242  Ohio  UNITED STATES |  | |
| Human Research Ethics Committee (TQEH/LMH/MH)  The Queen Elizabeth Hospital and Health Service Ethics of Human Research Committee, Woodville Rd  Woodville South 5011 South Australia  AUSTRALIA |  | |
| IEC of Haikou Municipal Hospital  No.43,Renmin Avenu,Haidiandao Haikou  570208  CHINA | IEC of Beijing Hospital No.1, Dahua Road Beijing  100730  CHINA | |
|  | Multicentricka eticka komise FN u sv. Anny v Brne  Pekarska 53 Brno  656 91  CZECH REPUBLIC | |
|  | CPP Sud-Est 3 CPP Lyon B Hôpital Hôtel Dieu Porte 16  1, place de l'Hôpital Lyon Cedex 02  69288  FRANCE | |
|  | Ethikkommision Universität Erlangen- Nürnberg  Ethik-Kommission der Medizinischen Fakultät Krankenhausstraße 12 Erlangen  91054  GERMANY | |
|  | Ethikkommision Universität Erlangen- Nürnberg  Ethik-Kommission der Medizinischen Fakultät Friedrich-Alexander- Krankenhausstraße 12 Erlangen  91054  GERMANY | |
|  | National Ethics Committee  284, Messoghion Avenue Cholargos Athens  15562  GREECE | |
|  | National Ethics Committee  284, Messoghion Avenue Cholargos Athens 15562  GREECE | |
|  | National Ethics Committee  284, Messoghion Avenue Cholargos Athens  15562  GREECE | |
| CEI Hospital Dr. Angel Leaño  Dr. Angel Leaño 500 Col. Los Robles Zapopan  45200  Jalisco MEXICO |  | |
|  | Catharina Ziekenhuis METC  Michelangelolaan 2 Eindhoven  5623 EJ NETHERLANDS | |
|  | National Ethics Committee for Clinical Trial of the Medicine 48, Av. Sanatescu Stefan str., Sector 1 Bucharest  011478  ROMANIA | |
|  | National Ethics Committee for Clinical Trial of the Medicine 48, Av. Sanatescu Stefan str., Sector 1 Bucharest  011478  ROMANIA | |
|  | National Ethics Committee for Clinical Trial of the Medicine 48, Av. Sanatescu Stefan str., Sector 1 Bucharest  011478  ROMANIA | |
| The South African Medical Association Block F Castle Walk Corporate Park Nossob Street Erasmuskloof Ext 3  Pretoria 0153 SOUTH AFRICA |  | |
| TC Sağlık Bakanlığı Türkiye İlaç ve Tıbbi Cihaz Kurumu , Klinik Araştırmalar Şube Müdürlüğü Sogutozu Mah. 2176, Sokak No:5 06520 Çankaya - Ankara TURKEY |  | |
| TC Sağlık Bakanlığı Türkiye İlaç ve Tıbbi Cihaz Kurumu , Klinik Araştırmalar Şube Müdürlüğü Sogutozu Mah. 2176, Sokak No:5 06520 Çankaya - Ankara TURKEY |  | |
| The Committee for Ethics Issues at the Institute of Neurology, Psychiatry and Narcology AMS Ukraine, 46, Acad. Pavlov Str., Kharkov, 61068, UKRAINE |  | |
| Local IEC of Chernivtsi Regional Regional Psychiatric Hospital MoH of Ukraine, 2, Musorgskogo Str., Chernivtsi, 50018, UKRAINE |  | |
| Schulman Associates Institutional Reveiw Board  4445 Lake Forest Drive Suite 300  Cincinnati 45242 Ohio  UNITED STATES |  | |
| Schulman Associates Institutional Reveiw Board  4445 Lake Forest Drive Suite 300 Cincinnati  45242 Ohio UNITED STATES |  | |
| Schulman Associates Institutional Reveiw Board  4445 Lake Forest Drive Suite 300  Cincinnati 45242  Ohio  UNITED STATES |  | |
| Western Institutional Review Board  3535 Seventh Avenue, SW  Olympia, WA 98502-  5010  UNITED STATES |  | |
| Schulman Associates Institutional Reveiw Board  4445 Lake Forest Drive Suite 300  Cincinnati 45242  Ohio  UNITED STATES |  | |
| Schulman Associates Institutional Reveiw Board  4445 Lake Forest Drive Suite 300  Cincinnati 45242 Ohio  UNITED STATES |  |  |
| St. Mary's of Michigan IRB  800 S. Washington Ave Saginaw  48601 Michigan UNITED STATES |  |  |
| Mount Sinai School of Medicine  Institutional Reveiw Board  101 East 101st , 1st floor Box 1075  New York, NY 10029 UNITED STATES |  |  |

**D. TENERE.**

| **INSTITUTIONAL REVIEW BOARD (IRB)/INDEPENDENT ETHICS COMMITTEE (IEC) Name and Address** | |
| --- | --- |
| **Local IRB/IEC** | **National IRB/IEC** |
|  | UZ Gent  De Pintelaan 185 Gent  9000  BELGIUM |
|  | UZ Gent  De Pintelaan 185 Gent  9000  BELGIUM |
|  | UZ Gent  De Pintelaan 185 Gent  9000  BELGIUM |
| Office of Research Ethics, The Univ of Western Ontario Room 5150 Support Services Building London  N6A 3K7  Ontario CANADA |  |
| Comite d'ethique de la recherche  143 Wolfe Street Levis  G6V 3Z1  Quebec CANADA |  |
| Health Research Ethics Authority  95 Bonaventure Avenue,  Suite 200 St. John'S A1B 2X5  Newfoundland and  Labrador CANADA |  |
| Eticka komise Vseobecne fakultni nemocnice v Praze Na Bojisti 1  III. patro Praha 2 12808  CZECH REPUBLIC | Eticka komise FNO a LFUP Olomouc  I. P. Pavlova 6 Olomouc 77520  CZECH REPUBLIC |
| Multicentricka eticka | Eticka komise FNO a |
| komise FNKV | LFUP Olomouc |
| Srobarova 50  Praha 10 | I. P. Pavlova 6 Olomouc |
| 10034 | 77520 |
| CZECH REPUBLIC | CZECH REPUBLIC |
|  | Eticka komise FNO a |
|  | LFUP Olomouc |
| Eticka komise nemocnice Jihlava Vrchlickeho 59  Jihlava | I. P. Pavlova 6 Olomouc 77520  CZECH REPUBLIC |
| 58601 |  |
| CZECH REPUBLIC |  |
|  | CPP Sud-Méditerranée 2  Hôpital Salvator  249 boulevard de Saint- Marguerite  Marseille Cedex 09  13274  FRANCE |
|  | CPP Sud-Méditerranée 2  Hôpital Salvator  249 boulevard de Saint- Marguerite  Marseille Cedex 09  13274  FRANCE |
|  | CPP Sud-Méditerranée 2  Hôpital Salvator  249 boulevard de Saint- Marguerite  Marseille Cedex 09  13274  FRANCE |
|  | CPP Sud-Méditerranée 2  Hôpital Salvator  249 boulevard de Saint- Marguerite  Marseille Cedex 09  13274  FRANCE |
|  | CPP Sud-Méditerranée 2  Hôpital Salvator  249 boulevard de Saint- Marguerite  Marseille Cedex 09  13274  FRANCE |
|  | Ethikkommission der Medizinischen Fakultät der Ruhr-Universität Bochum Gesundheitscampus 33 Bochum  44801  GERMANY |
|  | Ethikkommission der Medizinischen Fakultät der Ruhr-Universität Bochum Gesundheitscampus 33 Bochum  44801  GERMANY |
|  | Ethikkommission der Medizinischen Fakultät der Ruhr-Universität Bochum Gesundheitscampus 33 Bochum  44801  GERMANY |
|  | Ethikkommission der Medizinischen Fakultät der Ruhr-Universität Bochum Gesundheitscampus 33 Bochum  44801  GERMANY |
|  | Ethikkommission der Medizinischen Fakultät der Ruhr-Universität Bochum Gesundheitscampus 33 Bochum  44801  GERMANY |
|  | Ethikkommission der Medizinischen Fakultät der Ruhr-Universität Bochum Gesundheitscampus 33 Bochum  44801  GERMANY |
|  | Ethikkommission der Medizinischen Fakultät der Ruhr-Universität Bochum Gesundheitscampus 33 Bochum  44801  GERMANY |
|  | Ethikkommission der Medizinischen Fakultät der Ruhr-Universität Bochum Gesundheitscampus 33 Bochum  44801  GERMANY |
|  | Ethikkommission der Medizinischen Fakultät der Ruhr-Universität Bochum Gesundheitscampus 33 Bochum  44801  GERMANY |
|  | Ethikkommission der Medizinischen Fakultät der Ruhr-Universität Bochum Gesundheitscampus 33 Bochum  44801  GERMANY |
|  | Ethikkommission der Medizinischen Fakultät der Ruhr-Universität Bochum Gesundheitscampus 33 Bochum  44801  GERMANY |
|  | Ethikkommission der Medizinischen Fakultät der Ruhr-Universität Bochum Gesundheitscampus 33 Bochum  44801  GERMANY |
|  | Ethikkommission der Medizinischen Fakultät der Ruhr-Universität Bochum Gesundheitscampus 33 Bochum  44801  GERMANY |
|  | National Ethics Committee  284, Messoghion Avenue Cholargos Athens  15562  GREECE |
|  | National Ethics Committee  284, Messoghion Avenue Cholargos Athens  15562  GREECE |
|  | Medical Research Council, Ethics Committee for Clin. Pharm.  Klin. Farm. Etikai Bizottság, Arany János u. 6-8  Budapest 1051 HUNGARY |
|  | Medical Research Council, Ethics Committee for Clin. Pharm.  Klin. Farm. Etikai Bizottság, Arany János u. 6-8  Budapest 1051 HUNGARY |
|  | Medical Research Council, Ethics Committee for Clin. Pharm.  Klin. Farm. Etikai Bizottság, Arany János u. 6-8  Budapest 1051 HUNGARY |
|  | Medical Research Council, Ethics Committee for Clin. Pharm.  Klin. Farm. Etikai Bizottság, Arany János u. 6-8  Budapest 1051 HUNGARY |
|  | Medical Research Council, Ethics Committee for Clin. Pharm.  Klin. Farm. Etikai Bizottság, Arany János u. 6-8  Budapest 1051 HUNGARY |
|  | Medical Research Council, Ethics Committee for Clin. Pharm.  Klin. Farm. Etikai Bizottság, Arany János u. 6-8  Budapest 1051 HUNGARY |
|  | Medical Research Council, Ethics Committee for Clin. Pharm.  Klin. Farm. Etikai Bizottság, Arany János u. 6-8  Budapest 1051 HUNGARY |
|  | Medical Research Council, Ethics Committee for Clin. Pharm.  Klin. Farm. Etikai Bizottság, Arany János u. 6-8  Budapest 1051 HUNGARY |
|  | Medical Research Council, Ethics Committee for Clin. Pharm.  Klin. Farm. Etikai Bizottság, Arany János u. 6-8  Budapest 1051 HUNGARY |
| Ospedale San Raffaele Comitato Etico  Via Olgettina, 60 Milano  20132  Milano ITALY |  |
| Comitato Etico dell'Azienda Policlinico Umberto I di Roma Viale del Policlinico, 155 Roma  00155  Roma ITALY |  |
| AOU Policlinico Paolo Giaccone  Comitato per la Sperimentazione clinica dei Medicinali  Via del Vespro, 127 Palermo  90127  Palermo ITALY |  |
| Ospedale San Raffaele Comitato Etico  Via Olgettina, 60 Milano  20132  Milano ITALY |  |
| Comitato Etico Indip. Locale A.O. Policlinico Consorziale  Comitato Etico Indip. Locale A.O. Policlinico Consorziale  Piazza Giulio Cesare, 11 Bari  70124  Bari ITALY |  |
| Comitato Etico Interaziendale Corso Bramante, 88  A.O.“Città della Salute e della Scienza” di Torino Torino  10126  Torino ITALY |  |
| Comitato Etico Ospedale San Martino  Largo Rosanna Benzi, 10  Genova 16132  Genova ITALY |  |
| CE Indipendente AOU Cagliari  via Ospedale, 54 Cagliari  09124  Cagliari ITALY |  |
|  | Instytut Psychiatrii i Neurologii KOMISJA BIOETYCZNA  ul.Sobieskiego 9 Warszawa  02-957  Mazowieckie POLAND |
|  | Instytut Psychiatrii i Neurologii KOMISJA BIOETYCZNA  ul.Sobieskiego 9 Warszawa  02-957  Mazowieckie POLAND |
|  | Instytut Psychiatrii i Neurologii KOMISJA BIOETYCZNA  ul.Sobieskiego 9 Warszawa  02-957  Mazowieckie POLAND |
|  | Instytut Psychiatrii i Neurologii KOMISJA BIOETYCZNA  ul.Sobieskiego 9 Warszawa  02-957  Mazowieckie POLAND |
|  | CEIC Hospital Universitario Puerta de Hierro Majadahonda Joaquín Rodrigo, 2 Majadahonda  28222  Madrid SPAIN |
|  | CEIC Hospital Universitario Puerta de Hierro Majadahonda Joaquín Rodrigo, 2 Majadahonda  28222  Madrid SPAIN |
|  | CEIC Hospital Universitario Puerta de Hierro Majadahonda Joaquín Rodrigo, 2 Majadahonda  28222  Madrid SPAIN |
|  | CEIC Hospital Universitario Puerta de Hierro Majadahonda Joaquín Rodrigo, 2 Majadahonda  28222  Madrid SPAIN |
| Ethikkommission des Kantons St. Gallen Kantonsspital / Haus 57, Büro 007  Rorschacherstrasse 95 St. Gallen  9007  SWITZERLAND |  |
| Ethikkommission des Kantons St. Gallen Kantonsspital / Haus 57, Büro 007  Rorschacherstrasse 95 St. Gallen  9007  SWITZERLAND |  |
| Hôpital Fattouma Bourguiba  Rue Du 1er Juin 1995 Monastir  5000  TUNISIA |  |
|  | London - Hampstead REC  Northwick Park Hospital Watford Road  Harrow London HA1 3UJ  Greater London UNITED KINGDOM |
|  | London - Hampstead REC  Northwick Park Hospital Watford Road  Harrow London HA1 3UJ  Greater London UNITED KINGDOM |
|  | Eticka komise FNO a LFUP Olomouc  I. P. Pavlova 6 Olomouc 77520  CZECH REPUBLIC |
|  | Ethikkommission der Medizinischen Fakultät der Ruhr-Universität Bochum Gesundheitscampus 33 Bochum  44801  GERMANY |
|  | Ethikkommission der Medizinischen Fakultät der Ruhr-Universität Bochum Gesundheitscampus 33 Bochum  44801  GERMANY |
|  | National Ethics Committee  284, Messoghion Avenue Cholargos Athens  15562  GREECE |
| A. O. Istituti Clinici di Perfezionamento Comitato Etico  Via Castelvetro, 32 Milano  21054  Milano ITALY |  |
| Universitaria Ospeda Comitato Etico dell'Azienda Ospedaliero- Universitaria Ospedali Riuniti Umberto I - Lancisi - Salesi  Via Conca, 71 Ancona 60126  Ancona ITALY |  |
|  | CEIC Hospital Universitario Puerta de Hierro Majadahonda Joaquín Rodrigo, 2 Majadahonda  28222  Madrid SPAIN |
|  | CEIC Hospital  Universitario Puerta de Hierro Majadahonda  Joaquín Rodrigo, 2  Majadahonda  28222  Madrid  SPAIN |
|  | CEIC Hospital  Universitario Puerta de  Hierro Majadahonda  Joaquín Rodrigo, 2  Majadahonda  28222  Madrid  SPAIN |
| Institut National De  Neurologie  Service De Neurologie A  Rue Jebbari  La Rabta  Tunis  1007  TUNISIA |  |
